# Negative Cross Resistance Mediated by Co-Treated Bed Nets: A Potential Means of Restoring Pyrethroid-Susceptibility to Malaria Vectors

**DOI:** 10.1371/journal.pone.0095640

**Published:** 2014-05-01

**Authors:** Michael T. White, Dickson Lwetoijera, John Marshall, Geoffrey Caron-Lormier, David A. Bohan, Ian Denholm, Gregor J. Devine

**Affiliations:** 1 MRC Centre for Outbreak Analysis and Modelling, Imperial College, London, United Kingdom; 2 Ifakara Health Institute, Ifakara, Tanzania; 3 University of Nottingham, Sutton Bonington, Leicestershire, United Kingdom; 4 INRA, UMR 1347 Agroécologie, Pôle ECOLDUR, Dijon, France; 5 University of Hertfordshire, Hatfield, Hertfordshire, United Kingdom; 6 QIMR Berghofer Medical Research Institute, Brisbane, Australia; Institut Pasteur, France

## Abstract

Insecticide-treated nets and indoor residual spray programs for malaria control are entirely dependent on pyrethroid insecticides. The ubiquitous exposure of *Anopheles* mosquitoes to this chemistry has selected for resistance in a number of populations. This threatens the sustainability of our most effective interventions but no operationally practicable way of resolving the problem currently exists. One innovative solution involves the co-application of a powerful chemosterilant (pyriproxyfen or PPF) to bed nets that are usually treated only with pyrethroids. Resistant mosquitoes that are unaffected by the pyrethroid component of a PPF/pyrethroid co-treatment remain vulnerable to PPF. There is a differential impact of PPF on pyrethroid-resistant and susceptible mosquitoes that is modulated by the mosquito’s behavioural response at co-treated surfaces. This imposes a specific fitness cost on pyrethroid-resistant phenotypes and can reverse selection. The concept is demonstrated using a mathematical model.

## Introduction

A recent surge in effort and funding has led to the expansion of insecticide treated bed net (ITN) and indoor residual spray (IRS) programs in many parts of Africa and dramatic decreases in malaria transmission. Although four insecticide classes (carbamates, organophosphates, pyrethroids and the organochlorine DDT) are currently approved for IRS, the vast majority of spraying programs utilise synthetic pyrethroids. This is also the only insecticide class approved for use on ITNs [1]. The ubiquitous presence of pyrethroids in public health and the agricultural sector has resulted in strong selection pressure for mutations that confer resistance to pyrethroids in insect vectors of disease. In the absence of remedial measures, the impacts of this on malaria transmission can be severe [2], [3].

Pyrethroid resistance is widely reported in African malaria vectors [4] but there is little that can be done in response. There are few novel insecticidal products nearing commercialisation and the reassessment of old and previously resisted chemistries in new guises is now commonplace. A novel, resistance-beating combination of safe compounds with World Health Organisation (WHO) approval is therefore a timely and exciting proposition. We propose a mechanism to delay or reverse selection for pyrethroid resistance through a phenomenon called negative cross resistance (NCR) in which organisms resistant to one compound of a binary mixture are hyper-susceptible to the other. This imposes a fitness cost on the resistant genotype that can decrease the frequency of resistant alleles. This is distinct from the conventional use of binary mixtures and rotations where there is no hyper-sensitivity and whose role in resistance management is severely limited if the target pest has already developed resistance to either compound [5].

NCR has long been discussed by agricultural [6], [7] and public health entomologists [8] but it has largely eluded attempts at practical implementation. It remains an intriguing alternative to the “treadmill” approach of resistance management (the sequential replacement of one chemical class by another, as insects evolve a succession of protective mechanisms).

In our model, we exploit a potent chemosterilant (pyriproxyfen or PPF) and the differential behaviour of pyrethroid-resistant and susceptible mosquitoes at pyrethroid-treated surfaces. The model draws on the impacts of pyrethroids on susceptible and resistant insects and on recent proofs that PPF exposure dramatically reduces egg viability in *Anopheles gambiae* [9], [10].

### Assumptions

Our thesis requires unequivocal differences in the mortality and behaviour of pyrethroid-resistant and susceptible *Anopheles* mosquitoes when exposed to binary treatments of PPF and pyrethroids. Host-seeking or resting mosquitoes are more likely to be irritated, repelled or killed by co-treated surfaces if they are pyrethroid-susceptible. Conversely, pyrethroid-resistant insects are more likely to spend time resting or trying to feed at those surfaces. By surviving pyrethroid exposure they will pick up sterilising doses of PPF. This imposes a fitness cost on the pyrethroid-resistant phenotype. We call this phenomenon “behaviourally-mediated NCR”, since genotype selection results from a behavioural response rather than from any direct interaction between insecticides and physiological resistance mechanisms.

“Knock-down resistance” (kdr) is the most ubiquitous of the pyrethroid resistance mechanisms described for *An. gambiae* s.l. and other mosquito genera. It involves a modification of the pyrethroid target site and is often found in tandem with other detoxification mechanisms. It remains the best diagnostic for predicting pyrethroid-resistance [11]. The frequency of the allele in resistant field populations commonly ranges from 50–95% [12]–[15] and, unsurprisingly, resistant homozygotes can account for a large proportion of individuals [13], [16]. The mutation is incompletely recessive [17] and, in response to pyrethroids, heterozygotes (SR) suffer intermediate mortality to homozygous resistant (RR) and susceptible (SS) forms [13], [14]. Behavioural studies in the laboratory show that individuals carrying *kdr* alleles maintain contact with pyrethroid-treated surfaces for longer periods than susceptible insects, are less repelled and are more likely to blood-feed (i.e. through a treated net) than their susceptible counterparts. Heterozygotes tend to display intermediate behaviours [12], [13], [18], [19]. These impacts, in the presence of ITNs, have been widely demonstrated under field conditions and are most commonly recorded as differential blood-feeding success. Generally, SS insects are 2–5 fold less likely to feed than their SR and RR counterparts [20]–[24]. We exploit these behavioural differences to impose a PPF-mediated fitness-cost on pyrethroid-resistant mosquitoes exposed to PPF/pyrethroid co-treatments.

PPF is a juvenile hormone analogue with low toxicity to mammals. It inhibits metamorphosis and embryogenesis in several insects [25] and it is currently under evaluation by the World Health Organisation Pesticide Evaluation Scheme (WHOPES) as a component of a pyrethroid-treated bed net. It is approved as a mosquito larvicide and it may be suitable for autodissemination by mosquitoes for that purpose [26]. It is also a powerful chemosterilant. Exposure to PPF reduces the fecundity of adult female *An. gambiae* s.l. mosquitoes by reducing the number and viability of oviposited eggs. Ohashi *et al* [9] noted that the effects were dose-dependent and also reduced longevity. Harris *et al* [10] observed that *An. arabiensis* were completely sterilised for at least one gonotrophic cycle. Ohba et al [27] showed that both the fecundity and fertility of *Aedes albopictus* were affected when insects were exposed to PPF through a net while feeding on mice. These papers note that the sterilising impacts of PPF depend on the mosquito being exposed close to the time of feeding (the assumption being that PPF interferes with subsequent oogenesis and egg maturation) and suggest that co-treated bed nets may be an effective tool for exposing pyrethroid resistant mosquitoes to sterilising doses of PPF.

### Mathematical Models

We compare the reproductive fitness of *Anopheles gambiae* s.s. kdr susceptible (SS) and kdr homozygous resistant (RR) mosquitoes in the presence of PPF/pyrethroid co-treated surfaces. We first construct a static model to compare reproductive fitness in terms of the numbers of eggs oviposited by SS and RR mosquitoes. We then extend this to a dynamic mosquito population model with proportions of SS, SR and RR mosquitoes changing over time. We adapt a previously published model [28], [29] of the behavioural interactions between host seeking *Anopheles gambiae* mosquitoes and pyrethroid treated surfaces to estimate a mosquito’s daily mortality and feeding frequency. ITNs and IRS are assumed to have three effects on susceptible mosquitoes: (i) directly killing mosquitoes that land on treated surfaces; (ii) repelling and possibly diverting mosquitoes to an animal blood host due to either insecticide irritation or the physical barrier of the net; and (iii) lengthening the duration of the gonotrophic cycle leading to a reduced oviposition rate (by denying a blood meal). It is assumed that when kdr resistant mosquitoes encounter a pyrethroid treated net or surface, they (i) have a lower probability of being killed by pyrethroids; (ii) have a higher probability of successful feeding; and (iii) tend to be diverted by the physical barrier of the net as opposed to the irritant effect of the pyrethroids.

The key model parameters and the literature from which they are derived are defined in Table 1. The probabilities of a pyrethroid-susceptible mosquito feeding successfully (*s* = 0.03), being repelled (*r* = 0.56) or dying (*d* = 0.41) on exposure to an ITN are derived from empirical observations in experimental huts (26, 27). Resistant mosquitoes either die (*d* = 0.10) or are thwarted by the physical barrier of the net (*r* = 0.24) (27). The remainder is assumed to feed successfully. See Text S1, Tables S1 and S2 in Text S1, and Figure S1 for further explanation and illustration.

**Table 1:** Parameters for reproduction and interaction with pyrethroid/PPF co-treated surfaces.

Parameter	Description	Value		Reference
		pyrethroid resistance		
		susceptible	resistant	
*C*	ITN coverage (proportion of people under nets)	fixed	fixed	
*µ_M_*	daily non-insecticide mosquito mortality (day^−1^)	0.096	0.096	[30], [39]
ε	eggs per oviposition	74	74	[30]
δ	duration of gonotrophic cycle (days)	3	3	[40]
*Q_0_*	human blood index	0.90	0.90	[41]
φ	proportion of bites taken on humans while in bed	0.89	0.89	[42]
*s*	successful feeding with ITN	0.03	0.66	[43], [44]
*r*	cycle repeating probability for ITN	0.56	0.24	[43], [44]
*d*	insecticide mortality probability for ITN	0.41	0.10	[43], [44]
*p*_PPF_	probability of surviving contact with PPF treatedsurfaces *p*_PPF_ = *C Q*_0_φ(*s*+ *r*)	0*	model estimate	
*µ_M,ITN_ (C)*	daily mosquito mortality in the presence ofITNs (day^−1^) – see SI for details	model estimate	model estimate	
*f*_ITN_ = 1/δ_ITN_	blood feeding frequency in the presence ofITNs (day^−1^) – see SI for details	model estimate	model estimate	
	reduction in eggs: ITNs–0.001% w/v PPF	68%	68%	[9]
	reduction in eggs: ITNs–0.01 or 0.1% w/v PPF	100%	100%	[9]
	reduction in lifespan: ITNs–0.001% w/v PPF	38%	38%	[9]
	reduction in lifespan: ITNs–0.01% w/v PPF	55%	55%	[9]
	reduction in lifespan: ITNs–0.1% w/v PPF	75%	75%	[9]
	reduction in eggs: PPF treated surfaces	60–100%	60–100%	[10]
	reduction in lifespan: PPF treated surfaces	0%	0%	[10]

*Pyrethroid susceptible mosquitoes that contact a pyrethroid/PPF co-treated surface will be killed by the pyrethroid component. The survival of susceptible insects that avoid contact with the net (described by the terms *s*, *r* and *d*) is independent of this parameter.

The fitness of susceptible or resistant phenotypes is recorded as the expected number of eggs that a female mosquito will oviposit in her lifetime. A susceptible mosquito with daily mortality 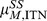, ovipositing *ε* eggs every 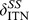 days, will oviposit an expected 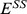 eggs over her lifetime, where
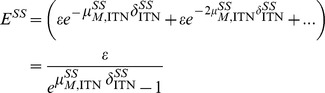



Without contacting PPF, pyrethroid-resistant mosquitoes will oviposit *ε* eggs every 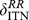 days and experience daily mortality 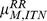. In the presence of PPF/pyrethroid co-treated nets at coverage *C*, resistant mosquitoes are exposed to PPF while attempting to feed with probability 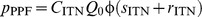. See SI text, section 2.2 for more detail. When exposed to PPF at co-treated surfaces, resistant mosquitoes will oviposit 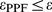 eggs and be subject to daily mortality 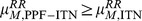. The expected number of eggs oviposited over the mosquito’s lifetime will be
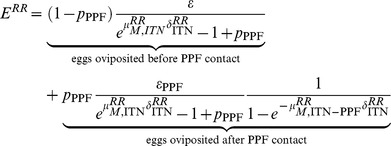



**Figure 1:**
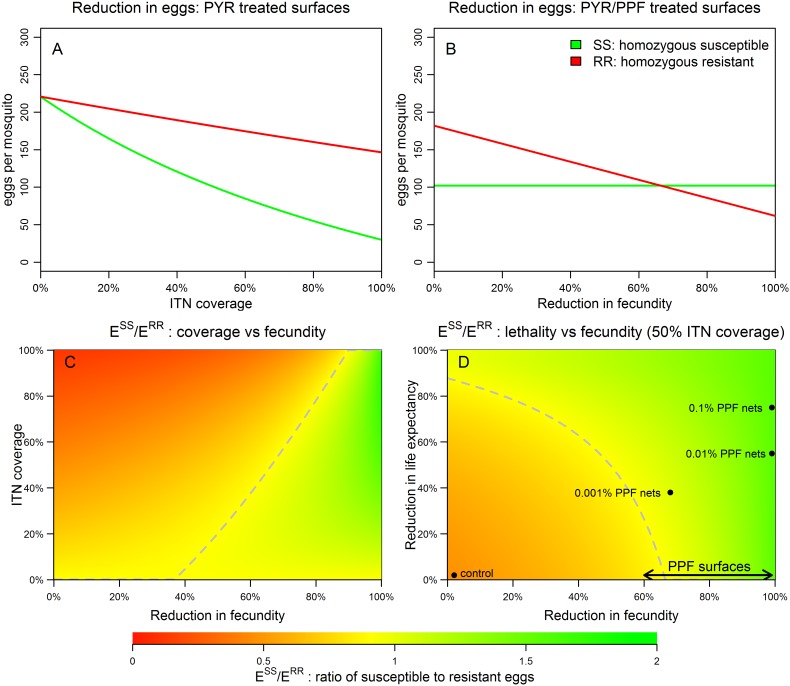
Reproductive fitness of pyrethroid susceptible (green) and resistant (red) mosquitoes in the presence of co-treated nets. Reduction in fecundity is defined as the proportional decrease in the number of eggs per oviposition. PYR = pyrethroid, PPF = pyriproxyfen. (**A**) Reduction in number of oviposited eggs with increasing coverage of ITNs. (**B**) Reduction in the number of oviposited eggs in presence of co-treated nets at 50% coverage. No reduction in life expectancy following PPF exposure is assumed. (**C**) Regions in parameter space where more eggs are oviposited by susceptible (green) than resistant (red) mosquitoes. No reduction in life expectancy following PPF exposure is assumed. (**D**) Regions in parameter space where more eggs are oviposited by susceptible (green) or resistant (red) mosquitoes at 50% ITN coverage. Reductions in fecundity and life expectancy observed by different concentration of PPF on bed nets by Ohashi *et al* [9] are represented as points. The range of reduction in fecundity seen by Harris *et al* [10] is represented by the black arrowed line. The dashed grey lines divide the parameter space into regions where susceptible mosquitoes are fitter than resistant mosquitoes (*E^SS^*>*E^RR^*), and where resistant mosquitoes are fitter than susceptible mosquitoes (*E^RR^*>*E^SS^*). The R code used to derive this figure is available as part of the supporting information (R code S1).

These equations describe the comparative reproductive fitness of homozygous pyrethroid-susceptible and resistant mosquitoes in terms of the numbers of eggs oviposited. See SI text section 3 and Figure S2 for more detail. The numbers and ratios of homozygous susceptible (SS) and resistant (RR) eggs that result from the presence of co-treated nets are illustrated in Figure 1. In situations where pyrethroid resistance is emerging, there will be a dynamic mix of SS, SR and RR mosquitoes. The model can also be extended to incorporate the number of eggs oviposited by heterozygous resistant mosquitoes and track the mixing of genotypes using a model of *An. gambiae* s.l. population dynamics [30]. Resistance is assumed (as is the case for kdr) to reflect a single locus, incompletely recessive allele [17] and we assume that SR mosquitoes have phenotypic properties intermediate between those of SS and RR. See SI text, Table S3 and Figure S4 for more detail on the dynamic model.

## Results

Increasing coverage of ITNs treated only with pyrethroids imparts a fitness advantage to pyrethroid-resistant mosquitoes. These are more likely to survive, blood-feed and oviposit. The consequent ratios of resistant: susceptible eggs will be large (Figure 1A). Co-treatment with PPF can reverse this advantage if the reduction in fecundity in pyrethroid-resistant mosquitoes contacting PPF is sufficiently large (Figure 1B).

At 50% coverage of co-treated ITNs, a 65% reduction in fecundity in exposed mosquitoes will reverse resistance selection by pyrethroids (Figure 1B). Higher levels of ITN coverage require increased impact of PPF to reverse that increased selection for resistance by pyrethroids (Figure 1C). Contact with PPF-treated surfaces may also shorten a mosquito’s lifespan and reduce the number of gonotrophic cycles and oviposition events [9]. This can affect disease transmission by reducing the time available for the incubation of viruses and parasites but, in this model we examine its additive effects on fitness costs in pyrethroid-resistant mosquitoes exposed to co-treated ITNs. The reductions in fecundity and life expectancy of mosquitoes exposed to nets treated with the formulations of pyriproxyfen used by Ohashi *et al* [9] and Harris *et al* [10] are highlighted in Figure 1D. These scenarios are not unrealistic: recent data shows that PPF can induce total sterilisation of mosquitoes using just 0.01% w/w on nets [9] or 3 mg/m^2^ on other substrates [10]. There is considerable potential to increase those doses.

Figure S3 extends the results of Figure 1 by illustrating the reversal of resistance selection at 30% and 80% ITN coverage. At low levels of ITN coverage, the emergence of pyrethroid resistance can be prevented either by modest reductions in fecundity or life expectancy. At higher levels of ITN coverage, reductions in lifespan alone are not sufficient to prevent the emergence of resistance, and large reductions in fecundity (>80%) are required.

**Figure 2:**
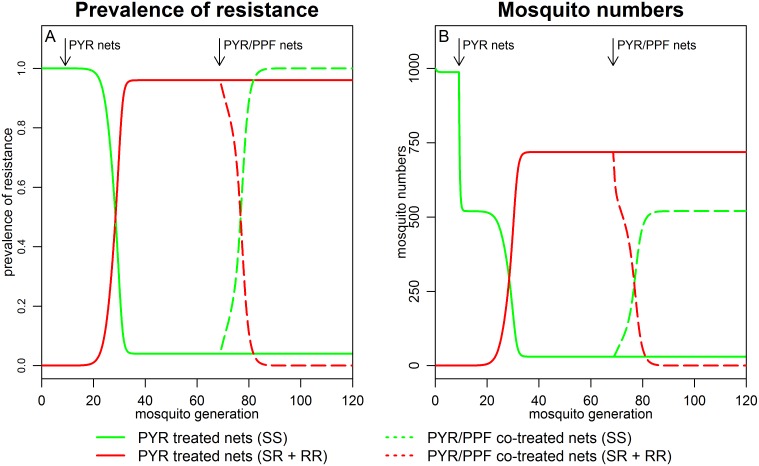
Emergence of pyrethroid resistance in the absence (solid lines) and presence of co-treated nets (dashed lines) at 50% coverage. PYR = pyrethroid, PPF = pyriproxyfen. Heterozygous resistant mosquitoes (SR) display behaviours intermediate to SS or RR genotypes, i.e. *h* = 0.5. (**A**) The introduction of ITNs treated with pyrethroids alone leads to the emergence of pyrethroid resistance but this is reversed by co-treating nets with PPF. The rate of reversal will depend on the percentage reduction in fecundity. (**B**) The introduction of ITNs causes a rapid decline in mosquito numbers, followed by the emergence of resistance and an increase in mosquito numbers. When resistance is reversed by the introduction of PPF, numbers remain suppressed as a consequence of mortality in the now largely pyrethroid-susceptible population. The initial frequency of homozygous resistant mosquitoes is assumed to be 10**^−^** ^5^. A mosquito generation is assumed to be the expected lifespan of the aquatic plus adult stages. The R code used to derive this figure is available as part of the supporting information (R code S2).

The emergence of pyrethroid resistance is likely to be a complex stochastic event, with unpredictable evolutionary scales. The deterministic model implemented here does not account for the emergence of novel resistance mechanisms or chance immigration of resistant mosquitoes, but it does illustrate the evolutionary outcomes that eventuate from selective pressure due to combinations of pyrethroids and PPF. It demonstrates a strong advantage to pyrethroid-susceptible genotypes. Figure 2A shows the emergence of pyrethroid resistance after the introduction of ITNs at 50% coverage and the subsequent reversal in allele frequency following the introduction of a PPF co-treatment that imposes a modest 68% decrease in fecundity (the reduction caused by exposure to 0.001% PPF exposure on nets [9]). Figure 2B shows the corresponding change in mosquito densities. In these scenarios, heterozygous resistant mosquitoes (SR) that encounter pyrethroid-treated surfaces display intermediate phenotypic behaviours in comparison to homozygous resistant (RR) or susceptible (SS) forms, i.e. the dominance co-efficient is *h* = 0.5. Figures S5 (*h* = 1) and S6 (*h* = 0.01) illustrate that the dominance co-efficient has relatively little impact on model outcomes at these levels of bed net coverage and imposed fitness cost.

## Discussion

The model-based investigations undertaken here suggest that the co-application of pyriproxyfen to pyrethroid treated nets or surfaces constitutes a plausible, practicable strategy for selecting against kdr resistant alleles. The technique that we exploit is distinct from the conventional use of binary mixtures and rotations where there is no hyper-sensitivity of resistant alleles and little advantage in terms of resistance management if the target pest has already developed resistance to either insecticide [5].

Although the sterilising effects of PPF might be used in a number of ways to suppress mosquito populations [31] we stress that, in this instance, the pyrethroid component of our proposed strategy is essential: it is the immediate, lethal impact of pyrethroids that permits the co-treated net to remain a successful disease intervention. In contrast, and unlike conventional toxins, PPF has little impact on mosquito longevity and exposed but infected mosquitoes will retain their capacity to survive the extrinsic incubation period and transmit disease. The purpose of the PPF component is to impose a cost on pyrethroid resistance, regain pyrethroid-susceptibility, and restore the overall effectiveness of ITNs.

Our model does not consider immigration of resistant alleles and makes the assumption that resistance is selected solely through interactions with treated bed nets. This reflects some empirical systems [32]–[34] but ignores the potential role of selection by pyrethroids used in agriculture and livestock [35]. It is expected, however, that resistant immigrants that encounter co-treated nets will be subject to the same fitness differential as resident insects. Other challenges to the feasibility of this resistance management approach might include avoidance of co-treated surfaces by RR or SR insects or recovery of fecundity with age. Neither scenario is likely. There is no evidence that PPF is repellent, even at high doses [36] and changes in fecundity are thought likely to be life-long following exposure to extremely practicable PPF concentrations [9], [10]. One other tangible threat to this chemically-based vector control solution is the appearance of novel resistance mechanisms (i.e. ones that reduce or negate the chemosterilant effect of PPF). In our modelled scenario, co-treatment offers some protection against that possibility: the pyrethroid-resistant individuals that encounter PPF will be strongly selected to evolve an additional PPF-resistance mechanism but pyrethroid-susceptible mosquitoes will be protected from PPF exposure, and hence from selection for PPF resistance, because of their responses to pyrethroids. Assuming random mating between genotypes, selection for PPF-resistant alleles should be constantly diluted by this pool of fully susceptible insects.

The modelled pyrethroid resistant mosquito population carries the incompletely recessive kdr resistance mechanism. This target-site mutation is an excellent diagnostic of pyrethroid-resistance [11] but additional mechanisms such as cytochrome P450 (CYP) are increasingly commonly described. Like kdr, these metabolic mechanisms are intermediately dominant in their heterozygote form [37] and individuals of species that exhibit mixtures of target site and metabolic mechanisms are observed to be spend a great deal of time in contact with pyrethroid-treated nets [23], [38]. It is likely therefore, that the behavioural differentials that we apply in our model are valid for most pyrethroid resistance mechanisms. Importantly, there is no evidence that CYP mechanisms alter the impact of pyriproxyfen’s chemosterilant effect.

An additional impact of PPF exposure, which we do not model, is PPF’s potential to be transferred from co-treated surfaces and to lethally affect juveniles developing in aquatic habitats. This phenomenon of “autodissemination” [26] may have profound impacts on population size but it will target aquatic environments irrespective of the juvenile phenotypes therein.

The co-application of pyrethroids and PPF may offer a powerful resistance management tool that complements the essential impacts of pyrethroids on mosquito population suppression and disease transmission. We offer an entirely different approach to the development of “resistance breaking” chemistries, which are simply new molecules as yet unresisted, or old molecules in new, more efficient guises. Solutions involving physiological NCR (in which pyrethroid-resistant populations are hyper-sensitive to a second insecticide but pyrethroid-susceptible populations are not) have no candidate molecules. Although we focus on a strategy where PPF is co-applied to pyrethroid treated nets, the model is broadly applicable to the same chemical combination deployed as an indoor residual spray. Our proposed strategy of “behaviourally-mediated NCR” utilises extant, registered and safe chemistries and merits urgent empirical investigation. Considerably more experimental data are needed to evaluate its practicality.

## Supporting Information

Figure S1 Flow chart of mosquito life cycle based on the diagram from Le Menach *et al* [7] and Griffin *et al* [6].
(TIF)

Figure S2 Flow chart depicting the life history and expected number of oviposited eggs of a pyrethroid-resistant mosquito. *M_n_* denotes a mosquito having completed *n* gonotrophic cycles. *M*_PPF,*n*_ denotes a mosquito that has completed *n* gonotrophic cycles and also been exposed to PPF. *p*_PPF_ is the probability that a mosquito contacts PPF at each feeding attempt. *q*_PPF_ = 1 - *p*_PPF_ is the probability that a mosquito avoids contact with PPF during a feeding attempt.
(TIF)

Figure S3 Comparison of the reproductive fitness of pyrethroid-susceptible and pyrethroid-resistant mosquitoes in the presence of co-treated pyrethroid/PPF nets at 30% and 80% coverage. Reduction in fecundity is defined as the proportional reduction in the number of eggs per oviposition. Red regions of parameter space represent scenarios where more eggs are oviposited by pyrethroid-resistant mosquitoes than pyrethroid-susceptible mosquitoes. Green regions of parameter space represent scenarios where more eggs are oviposited by pyrethroid-susceptible mosquitoes than pyrethroid- resistant mosquitoes. Yellow regions of parameter space represent scenarios where approximately the same number of eggs is oviposited by pyrethroid-susceptible mosquitoes and pyrethroid- resistant mosquitoes. Reductions in fecundity and life expectancy observed by different concentration of PPF on bed nets by Ohashi *et al* [11] are represented as points. The range of reduction in fecundity seen by Harris *et al* [12] is represented by the black arrowed line. The dashed grey lines divide the parameter space into regions where susceptible mosquitoes are fitter than resistant mosquitoes (*E^SS^*>*E^RR^*), and resistant mosquitoes are fitter than susceptible mosquitoes (*E^RR^*>*E^SS^*). The R code for generating this figure is included as a supporting file (R code S1).
(TIF)

Figure S4 Flow chart for the numbers of aquatic stages (early and late larval instars and pupae) and adult mosquitoes stratified by gonotrophic cycle and PPF exposure status.
(TIF)

Figure S5 Emergence of pyrethroid resistance in the absence (solid lines) and presence of co-treated nets (dashed lines) at 50% coverage. PYR = pyrethroid, PPF = pyriproxyfen. It is assumed that heterozygous resistant mosquitoes (SR) have the same phenotypic behaviour as homozygous resistant mosquitoes (RR), i.e. *h* = 1.
(TIF)

Figure S6 Emergence of pyrethroid resistance in the absence (solid lines) and presence of co-treated nets (dashed lines) at 50% coverage. PYR = pyrethroid, PPF = pyriproxyfen. It is assumed that heterozygous resistant mosquitoes (SR) have the similar phenotypic behaviour as homozygous susceptible mosquitoes (SS), i.e. *h* = 0.1.
(TIF)

R Code S1 Code is for Figure 1 and Figure S3.
(DOCX)

R Code S2 Code is for Figure 2.
(DOCX)

Text S1 Contains Table S1, parameters describing the behaviour and life history of *An. gambiae* s. s. mosquitoes. Table S2, parameters describing interactions between a mosquito and an insecticide-treated net. Table S3, notation, definition and values of the variables and parameters for the model of *A. gambiae* population dynamics. All parameter values are taken from White *et al* [2].
(DOCX)
