# Supplementary figures and images for: Negative Cross Resistance Mediated by Co-Treated Bed Nets: A Potential Means of Restoring Pyrethroid-Susceptibility to Malaria Vectors
Source: PLoS One. 2014 May 1;9(5):e95640. doi: 10.1371/journal.pone.0095640 (PMC4006834; doi:10.1371/journal.pone.0095640)

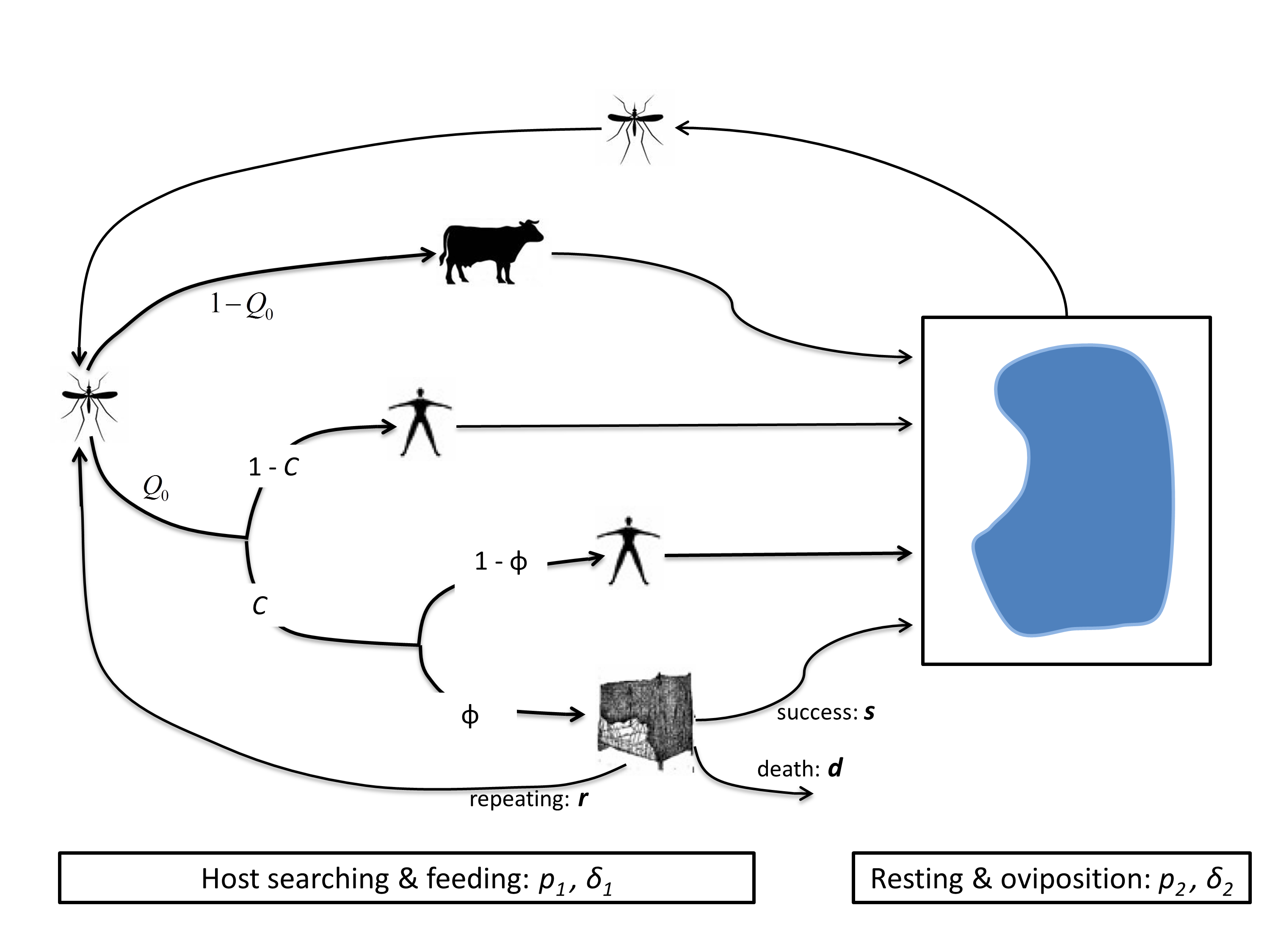

Supplement: Figure S1 — Flow chart of mosquito life cycle based on the diagram from Le Menach et al [7] and Griffin et al [6]. (TIF) [file pone.0095640.s001.tif]

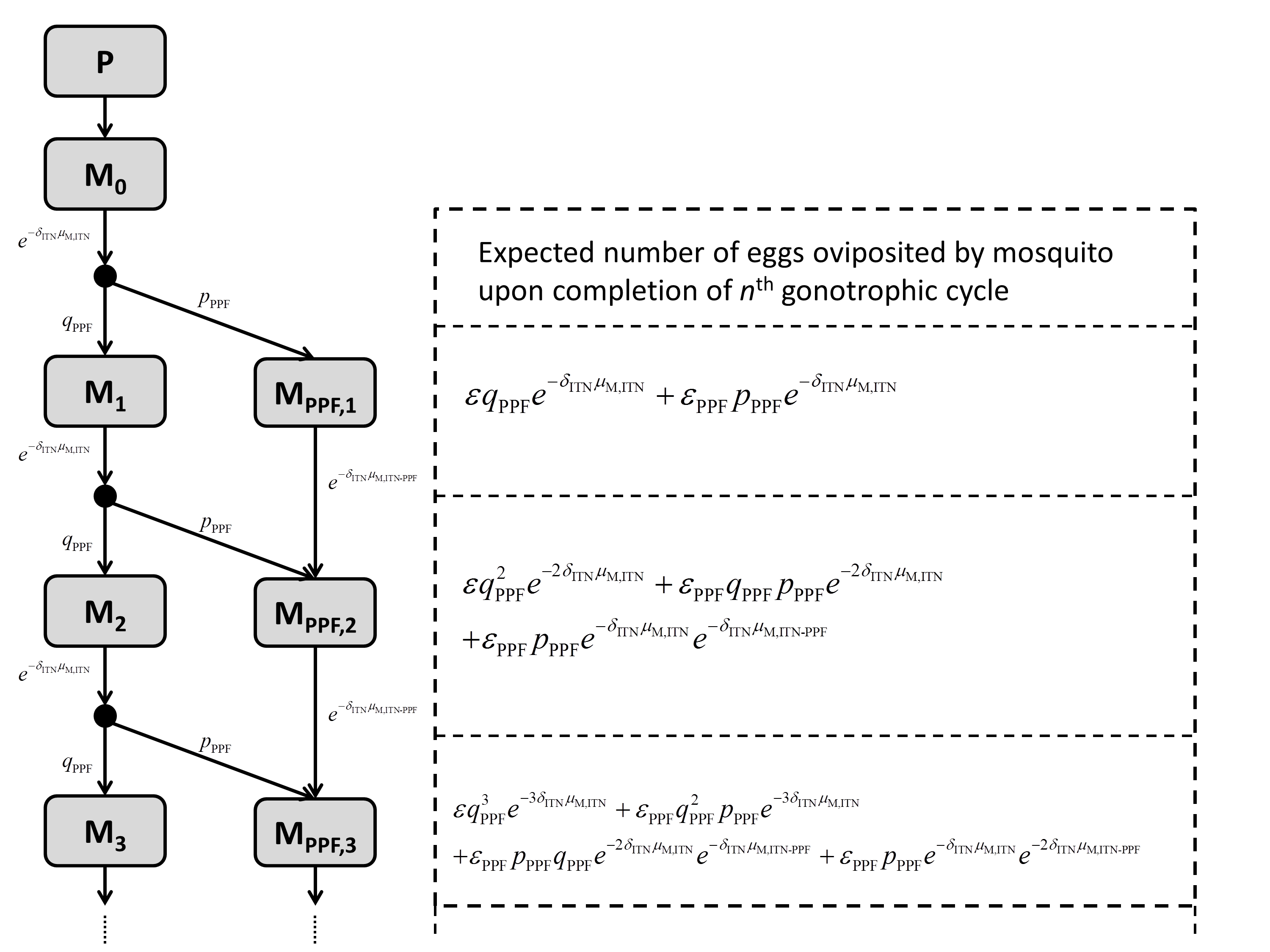

Supplement: Figure S2 — Flow chart depicting the life history and expected number of oviposited eggs of a pyrethroid-resistant mosquito. Mn denotes a mosquito having completed n gonotrophic cycles. M PPF,n denotes a mosquito that has completed n gonotrophic cycles and also been exposed to PPF. p PPF is the probability that a mosquito contacts PPF at each feeding attempt. q PPF = 1 - p PPF is the probability that a mosquito avoids contact with PPF during a feeding attempt. (TIF) [file pone.0095640.s002.tif]

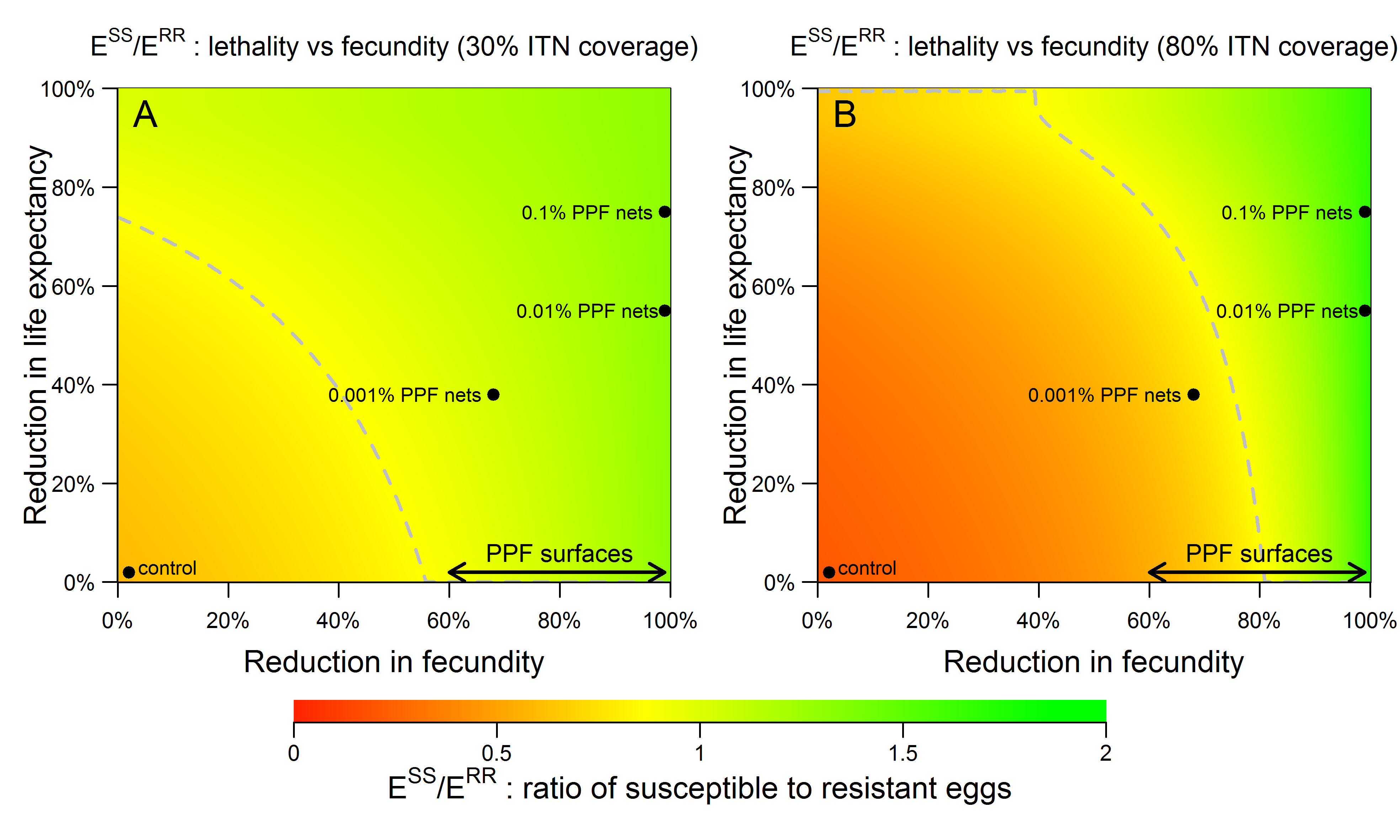

Supplement: Figure S3 — Comparison of the reproductive fitness of pyrethroid-susceptible and pyrethroid-resistant mosquitoes in the presence of co-treated pyrethroid/PPF nets at 30% and 80% coverage. Reduction in fecundity is defined as the proportional reduction in the number of eggs per oviposition. Red regions of parameter space represent scenarios where more eggs are oviposited by pyrethroid-resistant mosquitoes than pyrethroid-susceptible mosquitoes. Green regions of parameter space represent scenarios where more eggs are oviposited by pyrethroid-susceptible mosquitoes than pyrethroid- resistant mosquitoes. Yellow regions of parameter space represent scenarios where approximately the same number of eggs is oviposited by pyrethroid-susceptible mosquitoes and pyrethroid- resistant mosquitoes. Reductions in fecundity and life expectancy observed by different concentration of PPF on bed nets by Ohashi et al [11] are represented as points. The range of reduction in fecundity seen by Harris et al [12] is represented by the black arrowed line. The dashed grey lines divide the parameter space into regions where susceptible mosquitoes are fitter than resistant mosquitoes (ESS>ERR), and resistant mosquitoes are fitter than susceptible mosquitoes (ERR>ESS). The R code for generating this figure is included as a supporting file (R code S1). (TIF) [file pone.0095640.s003.tif]

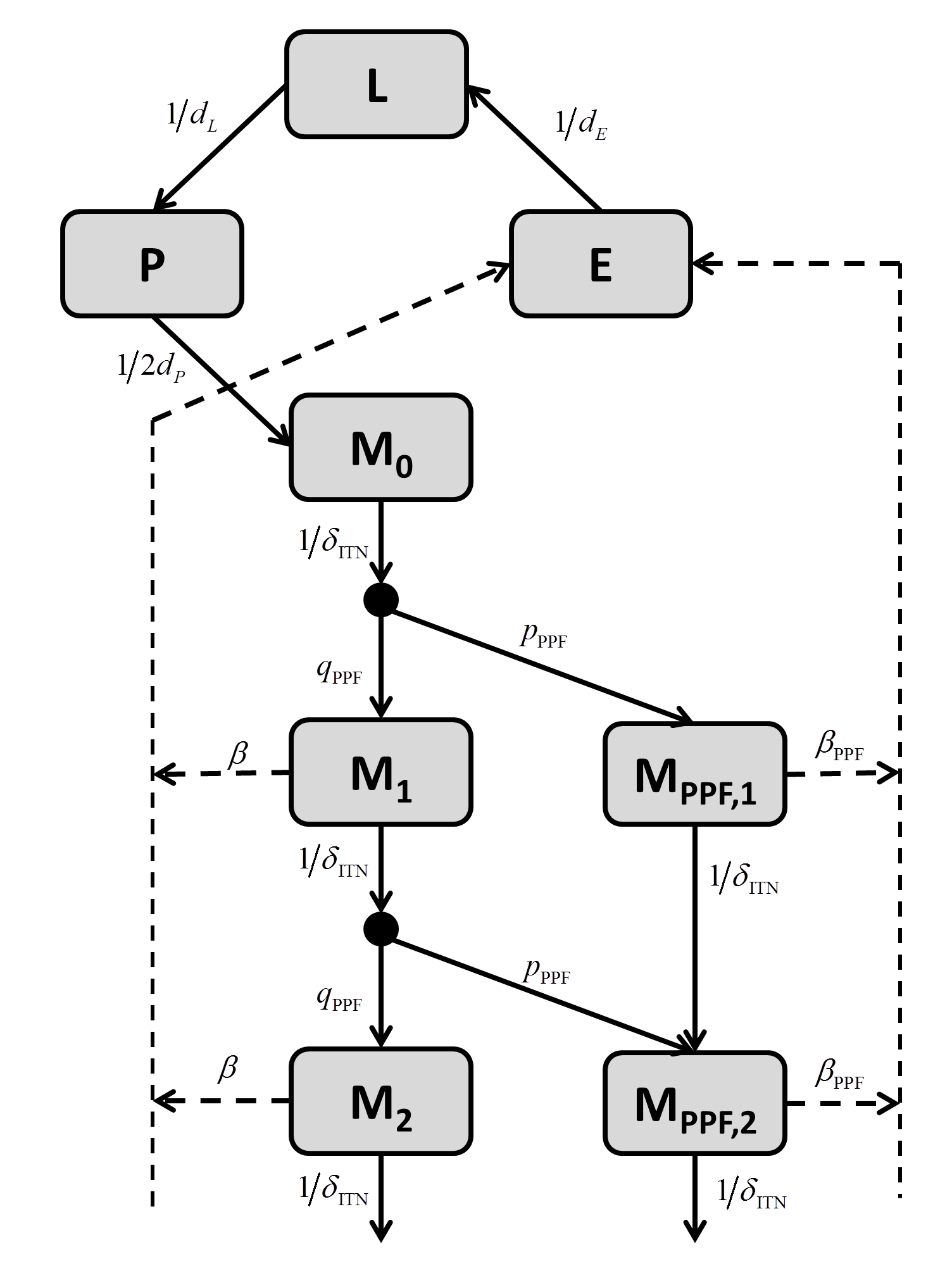

Supplement: Figure S4 — Flow chart for the numbers of aquatic stages (early and late larval instars and pupae) and adult mosquitoes stratified by gonotrophic cycle and PPF exposure status. (TIF) [file pone.0095640.s004.tif]

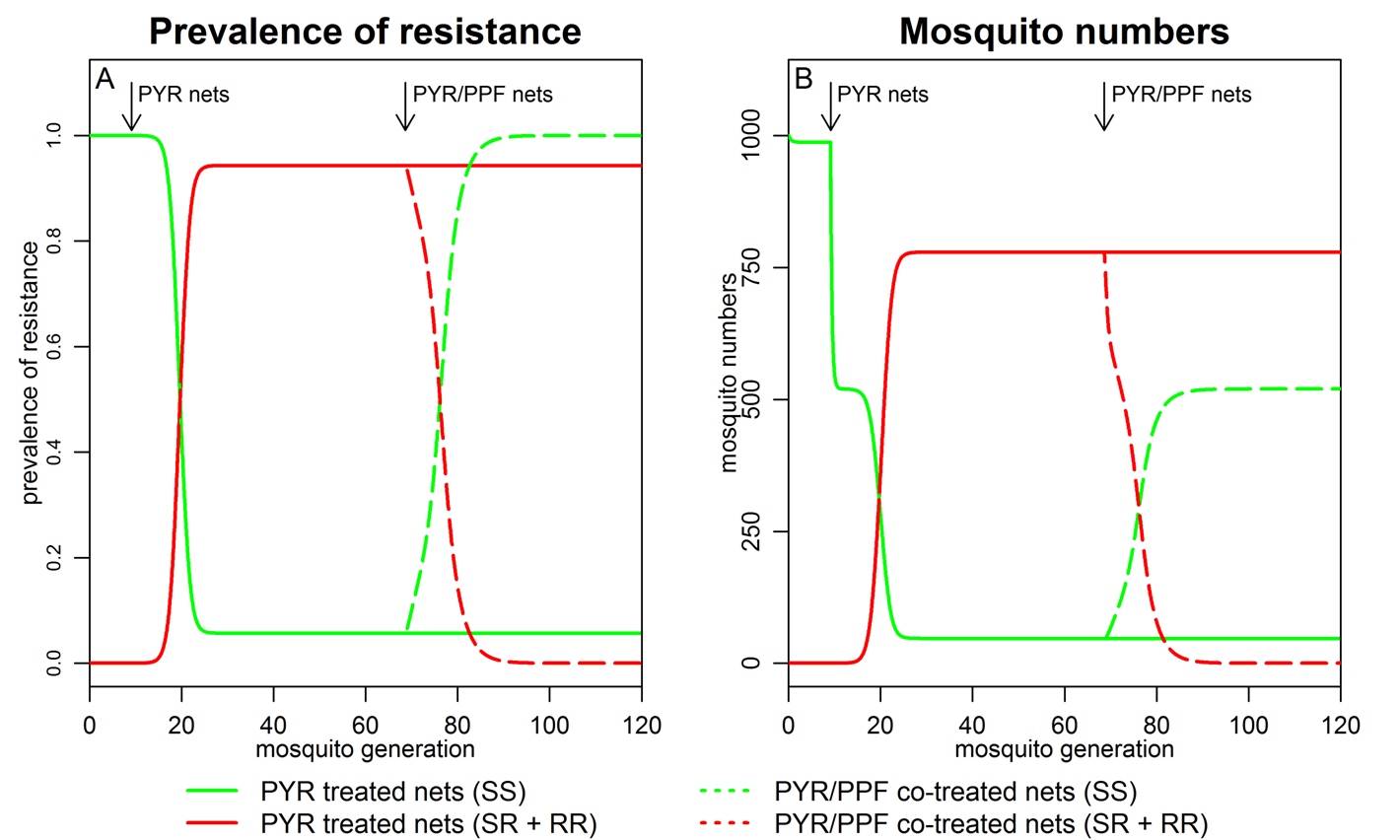

Supplement: Figure S5 — Emergence of pyrethroid resistance in the absence (solid lines) and presence of co-treated nets (dashed lines) at 50% coverage. PYR = pyrethroid, PPF = pyriproxyfen. It is assumed that heterozygous resistant mosquitoes (SR) have the same phenotypic behaviour as homozygous resistant mosquitoes (RR), i.e. h = 1. (TIF) [file pone.0095640.s005.tif]

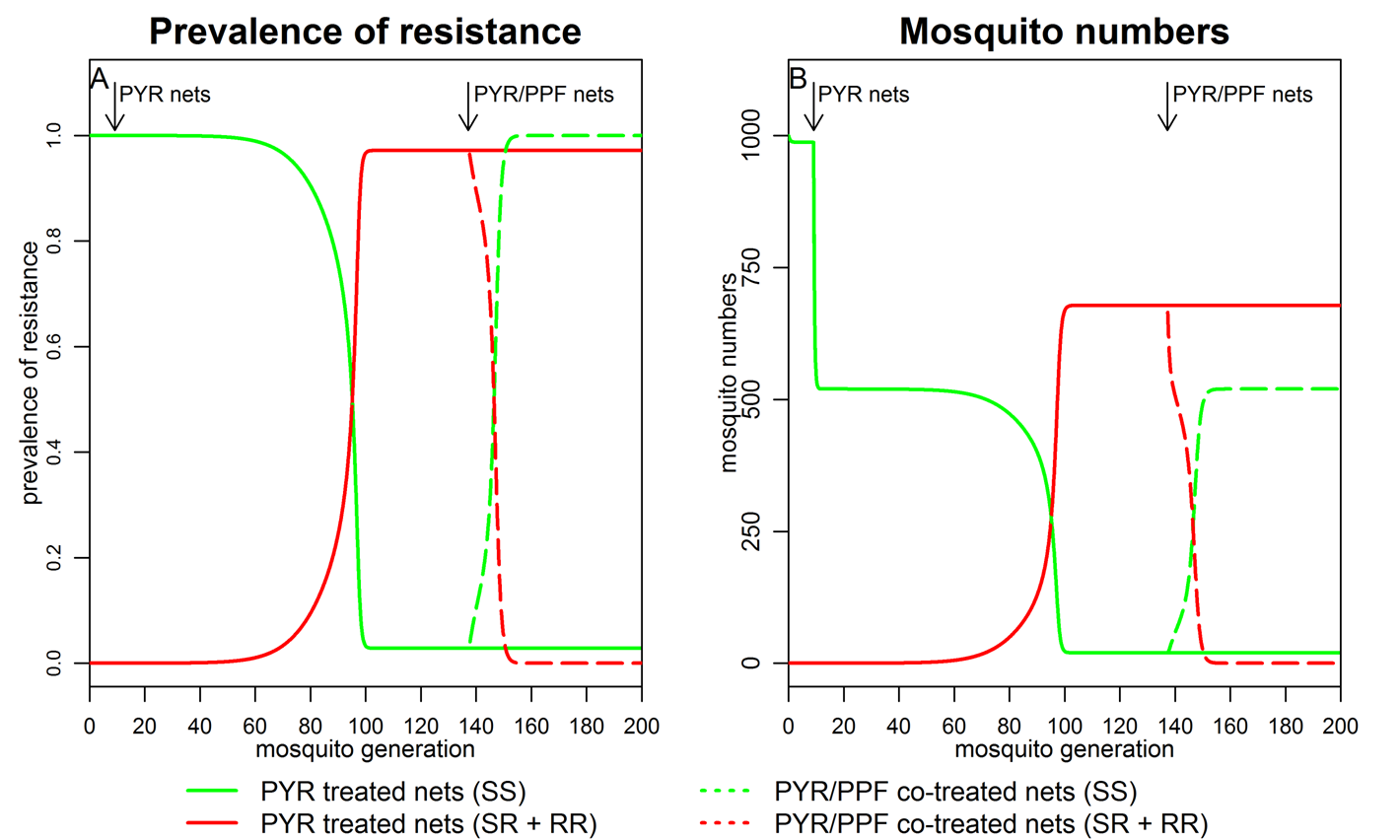

Supplement: Figure S6 — Emergence of pyrethroid resistance in the absence (solid lines) and presence of co-treated nets (dashed lines) at 50% coverage. PYR = pyrethroid, PPF = pyriproxyfen. It is assumed that heterozygous resistant mosquitoes (SR) have the similar phenotypic behaviour as homozygous susceptible mosquitoes (SS), i.e. h = 0.1. (TIF) [file pone.0095640.s006.tif]
