# Supplementary material for: Negative Cross Resistance Mediated by Co-Treated Bed Nets: A Potential Means of Restoring Pyrethroid-Susceptibility to Malaria Vectors
Source: PLoS One. 2014 May 1;9(5):e95640. doi: 10.1371/journal.pone.0095640 (PMC4006834; doi:10.1371/journal.pone.0095640)
Supplement: R Code S1 — Code is for Figure 1 and Figure S3. (DOCX) [file pone.0095640.s007.docx]

########################################################################################################

########################################################################################################

## Supporting File: R code for Figure 1 (main text) and Figure S3 (supporting information) ##

## ##

## Please feel free to use and modify with proper citation. ##

## For any questions or comments, please contact ##

## ##

## ##

## Dr Michael White ##

## MRC Centre for Outbreak Analysis and Modelling ##

## Department of Infectious Disease Epidemiology ##

## Imperial College London ##

## m.white08@imperial.ac.uk ##

## ##

########################################################################################################

########################################################################################################

library(fields)

###########################

## Entomological parameters describing

## mosquito life history and

## interaction with ITNs

mu_M0 <- 0.096 ## death rate of adult female mosquitoes - no nets

beta <- 21.19 ## daily ovipositon rate

delta <- 3 ## duration of gonotrophic cycle

eps0 <- beta*( exp(mu_M0*delta) - 1 )/mu_M0 ## number of eggs per oviposition

Q_0 <- 0.90 ## human blood index

phi <- 0.89 ## endophagy in bed

tau_1 <- 0.69 ## time spent searching for blood meal

tau_2 <- delta - tau_1 ## time spent resting and ovipositing

###########################################

## Genotype specific mosquito parameters

## for interactions with ITNs

mu_M <- c(mu_M0, mu_M0, mu_M0) ## genotype specific mortality (SS, SR, RR)

h = 0.5 ## heterozygosity: h=0 => SR=SS, h=1 => SR=RR

r_ss = 0.56 ## Prob SS mosquito repeats upon encountering ITN

s_ss = 0.03 ## Prob SS mosquito feeds successfully upon encountering ITN

d_ss = 0.41 ## Prob SS mosquito dies upon encountering ITN

r_rr = 0.24 ## Prob RR mosquito repeats upon encountering ITN

d_rr = 0.1 ## Prob RR mosquito feeds successfully upon encountering ITN

s_rr = 1 - r_rr - d_rr ## Prob RR mosquito dies upon encountering ITN

r_sr = h*r_rr + (1-h)*r_ss ## Prob SR mosquito repeats upon encountering ITN

d_sr = h*d_rr + (1-h)*d_ss ## Prob SR mosquito feeds successfully upon encountering ITN

s_sr = 1 - r_sr - d_sr ## Prob SR mosquito dies upon encountering ITN

r_ITN <- c(r_ss, r_sr, r_rr)

d_ITN <- c(d_ss, d_sr, d_rr)

s_ITN <- c(s_ss, s_sr, s_rr)

##############################################################

## eggs_model: calculate expected number of eggs oviposited ##

## over a female mosquito's lifetime ##

##############################################################

eggs_model <- function( P_eggs, P_life, itn_cov ){

###########################################

## PART 1: Effect of PYR nets on mosquitoes

W <- 1 - Q_0*itn_cov*phi*(1 - s_ITN) ## probability of mosquito succeeding in feeding in a single attempt

Z <- Q_0*itn_cov*phi*r_ITN ## probability of mosquito repeating in a single attempt

f_ITN <- 1/( tau_1/(1-Z) + tau_2 ) ## rate at which mosquito feeds

p_10 <- exp(-mu_M*tau_1) ## probability of mosquito dying during feeding at zero ITN coverage

p_2 <- exp(-mu_M*tau_2) ## probability of mosquito dying during resting and oviposition

p_1 <- p_10*W/(1-Z*p_10) ## probability of mosquito dying during feeding

mu_M_ITN <- - f_ITN*log(p_1*p_2) ## daily mosquito mortality

delta_ITN <- 1/f_ITN ## length of gonotrophic cycle - extended due to extra host seeking time

S_m = exp( -mu_M_ITN*delta_ITN ) ## Probabiliy of surviving a gonotrophic cycle

###########################################

## PART 2: Effect of PPF on mosquitoes

P_ppf = Q_0*itn_cov*phi*(r_ITN + s_ITN) ## Probability of contacting PPF during a single feeding attempt

P_ppf[1] = 0 ## SS mosquitoes don't contact PPF

Q_ppf = 1 - P_ppf ## Probability of not contacting PPF

###############################################

## PART 3: Effect of PYR/PPF nets on mosquitoes

mu_M_ppf <- mu_M/P_life ## adult mosquito mortality (reduced due to PPF)

p_10_ppf <- exp(-mu_M_ppf*tau_1) ## probability of mosquito dying during feeding at zero ITN coverage

p_2_ppf <- exp(-mu_M_ppf*tau_2) ## probability of mosquito dying during resting and oviposition

p_1_ppf <- p_10*W/(1-Z*p_10_ppf) ## probability of mosquito dying during feeding

mu_M_ppf_ITN <- - f_ITN*log(p_1_ppf*p_2_ppf) ## mortality due to PYR/PPF nets

S_x = exp( - mu_M_ppf_ITN*delta_ITN ) ## Probabiliy of surviving a gonotrophic cycle

###############################################

## PART 4: Output expected number of lifetime eggs

eggs <- eps0*S_m*Q_ppf/(1-S_m*Q_ppf) + eps0*P_eggs*( S_m*P_ppf/(1-S_m*Q_ppf) )/(1-S_x)

eggs

}

###############################################

## eggs_test: check whether SS mosquitoes or ##

## RR mosquitoes ovipoist more eggs ##

###############################################

eggs_test <- function( P_eggs, P_life, itn_cov ){

eggs = eggs_model( P_eggs, P_life, itn_cov )

out <- 0

if( eggs[1] > eggs[3] ){

out <- 1

}

out

}

######################################################

## eggs_ratio: calculate the ratio of SS to RR eggs ##

######################################################

eggs_ratio <- function( P_eggs, P_life, itn_cov ){

eggs = eggs_model( P_eggs, P_life, itn_cov )

eggs[1]/eggs[3]

}

step_size <- 0.001

#################

## ##

## SIMULATIONS ##

## PANEL A ##

## ##

#################

itn_cov_seq <- seq( from=0, to=1, by=step_size) ## Sequence for varying ITN coverage

###################################################################

## Calculate number of eggs by SS and RR at varying ITN coverage

eggs_ITN_SS <- rep(NA, length(itn_cov_seq))

eggs_ITN_RR <- rep(NA, length(itn_cov_seq))

for(i in 1:length(eggs_ITN_SS)){

temp <- eggs_model(1, 1, itn_cov_seq[i])

eggs_ITN_SS[i] <- temp[1]

eggs_ITN_RR[i] <- temp[3]

}

#################

## ##

## SIMULATIONS ##

## PANEL B ##

## ##

#################

fecund_seq <- seq( from=step_size, to=1, by=step_size) ## Sequence for varying fecundity reductions

###################################################################

## Calculate number of eggs by SS and RR at varying fecundity reductions

eggs_fecund_SS <- rep(NA, length(fecund_seq))

eggs_fecund_RR <- rep(NA, length(fecund_seq))

for(i in 1:length(fecund_seq)){

temp <- eggs_model(fecund_seq[i], 1, 0.5)

eggs_fecund_SS[i] <- temp[1]

eggs_fecund_RR[i] <- temp[3]

}

#################

## ##

## SIMULATIONS ##

## PANEL C ##

## ##

#################

fecund_seq <- seq(from=0, to=1-step_size, by=step_size) ## sequence for redcution in fecundity

itn_seq <- seq(from=step_size, to=1, by=step_size) ## sequence for ITN coverage

###################################################################

## Test parameter spce to see whether SS or RR mosquitoes oviposit more eggs

eggs_mat1 <- matrix(NA, nrow=length(itn_seq), ncol=length(fecund_seq))

for(i in 1:length(itn_seq)){

for(j in 1:length(fecund_seq)){

eggs_mat1[i,j] <- eggs_ratio( 1-fecund_seq[i], 1, itn_seq[j] )

}

}

SR_divide_C <- rep(NA, length(fecund_seq))

for(i in 1:length(SR_divide_C)){

SR_divide_C[i] <- itn_seq[which.min((eggs_mat1[i,]-1)^2)]

}

SR_divide_C[which.max(SR_divide_C):length(SR_divide_C)] <- 1

#################

## ##

## SIMULATIONS ##

## PANEL D ##

## ##

#################

fecund_seq <- seq(from=0, to=1-step_size, by=step_size) ## sequence for redcution in fecundity

life_seq <- seq(from=0, to=1-step_size, by=step_size) ## sequence for redcution in life expectancy

###################################################################

## Test parameter spce to see whether SS or RR mosquitoes oviposit more eggs

eggs_mat2 <- matrix(NA, nrow=length(fecund_seq), ncol=length(life_seq))

for(i in 1:length(fecund_seq)){

for(j in 1:length(life_seq)){

eggs_mat2[i,j] <- eggs_ratio( 1-fecund_seq[i], 1-life_seq[j], 0.5 )

}

}

SR_divide_D <- rep(NA, length(fecund_seq))

for(i in 1:length(SR_divide_D)){

SR_divide_D[i] <- life_seq[which.min((eggs_mat2[i,]-1)^2)]

}

################################################

## Create colour gradient

Ncol = 1000

eggs_max <- 2 # max( eggs_mat1, eggs_mat2)

eggs_min <- 0 # min( eggs_mat1, eggs_mat2)

N_red <- round( Ncol*( 1-eggs_min )/( eggs_max - eggs_min ) )

N_green <- round( Ncol*( eggs_max-1 )/( eggs_max - eggs_min ) )

col_red_yellow <- rainbow(N_red, start=0, end=1/6)

col_yellow_green <- rainbow(N_green, start=1/6, end=2/6)

colours <- c(col_red_yellow, col_yellow_green)

####################################################

####################################################

## ##

## ##### #### #### ## ## ##### ##### ## ##

## ## ## ## ## ## ## ## ## ### ##

## #### ## ## ### ## ## ##### #### ## ##

## ## ## ## ## ## ## ## ## ## ## ##

## ## #### #### #### ## ## ##### #### ##

## ##

####################################################

####################################################

tiff(file="Figure1.tif", width=20, height=18, units="cm", res=500)

lay.mat <- rbind( c(1,2), c(3,4), c(5,5) )

layout(lay.mat, heights=c(1,1,0.2))

layout.show(5)

par(mar=c(3,3,3,1.0))

par(mgp=c(2,0.75,0))

main.size = 1.4

axis.size = 1

lab.size = 1.25

line.size = 2

#############

## ##

## PANEL A ##

## ##

###########################################################

## Expected number of eggs as a function of ITN coverage ##

###########################################################

##################################

## Plot eggs vs ITN coverage

plot(x=itn_cov_seq, y=eggs_ITN_SS,

type='l', lwd=line.size, col="green",

ylim=c(0,300), xlim=c(0,1),

xlab="ITN coverage", ylab="eggs per mosquito",

main=expression(paste("Reduction in eggs: PYR treated surfaces")),

xaxt="n", xaxs="i",

cex.main=main.size, cex.lab=lab.size, cex.axis=axis.size )

points(x=itn_cov_seq, y=eggs_ITN_RR,

type='l', lwd=line.size, col="red")

axis(1, at=seq(0,1, by=0.2), lab=c("0%", "20%", "40%", "60%", "80%", "100%"), las=TRUE,

cex.axis=axis.size)

text(x=0.05, y=0.95*300, labels="A", cex=1.5)

#############

## ##

## PANEL B ##

## ##

####################################################################

## Expected number of eggs laid at 50% ITN coverage as a function ##

## of reduction in fecundity ##

####################################################################

##################################

## Plot eggs vs fecundity reductions

plot(x=1-fecund_seq, y=eggs_fecund_SS,

type='l', lwd=line.size, col="green",

ylim=c(0,300), xlim=c(0,1),

xaxt="n", xaxs="i",

xlab="Reduction in fecundity", ylab="eggs per mosquito",

main=expression(paste("Reduction in eggs: PYR/PPF treated surfaces")),

cex.main=main.size, cex.lab=lab.size, cex.axis=axis.size )

points(x=1-fecund_seq, y=eggs_fecund_RR,

type='l', lwd=line.size, col="red")

axis(1, at=seq(0,1, by=0.2), lab=c("0%", "20%", "40%", "60%", "80%", "100%"), las=TRUE,

cex.axis=axis.size)

text(x=0.05, y=0.95*300, labels="B", cex=1.5)

legend(x='topright', legend=c("SS: homozygous susceptible", "RR: homozygous resistant"),

fill = c("green", "red"),

cex=1.25, bty="n", border=NULL )

#############

## ##

## PANEL C ##

## ##

##################################################################

## Comparison of egg reduction vs. itn coverage parameter space ##

## for regions of suppression of resistance ##

##################################################################

par(mar=c(4,4,3,1))

par(mgp=c(2.25,0.75,0))

##########################

## Plot output

image( eggs_mat1,

xlab="Reduction in fecundity", ylab="ITN coverage",

main=expression(paste( "E"^"SS", "/E"^"RR", " : coverage vs fecundity")),

col=colours, zlim=c(eggs_min, eggs_max),

xaxt="n", yaxt="n", xaxs="i", yaxs="i",

cex.main=main.size, cex.lab=lab.size, cex.axis=axis.size )

points(x=fecund_seq, y=SR_divide_C, type='l', lty="dashed", col="grey", lwd=2)

axis(1, at=seq(0,1, by=0.2), lab=c("0%", "20%", "40%", "60%", "80%", "100%"), las=TRUE,

cex.axis=0.9*axis.size)

axis(2, at=seq(0,1, by=0.2), lab=c("0%", "20%", "40%", "60%", "80%", "100%"), las=TRUE,

cex.axis=0.9*axis.size)

text(x=0.05, y=0.95, labels="C", cex=1.5)

#############

## ##

## PANEL D ##

## ##

####################################################################

## Comparison of egg reduction vs. life reduction parameter space ##

## for regions of suppression of resistance ##

####################################################################

par(mar=c(4,4,3,1))

par(mgp=c(2.25,0.75,0))

##########################

## Plot output

image( eggs_mat2,

xlab="Reduction in fecundity", ylab="Reduction in life expectancy",

main=expression(paste( "E"^"SS", "/E"^"RR", " : lethality vs fecundity (50% ITN coverage)")),

col=colours, zlim=c(eggs_min, eggs_max),

xaxt="n", yaxt="n", xaxs="i", yaxs="i",

cex.main=main.size, cex.lab=lab.size, cex.axis=axis.size )

points(x=fecund_seq, y=SR_divide_D, type='l', lty="dashed", col="grey", lwd=2)

axis(1, at=seq(0,1, by=0.2), lab=c("0%", "20%", "40%", "60%", "80%", "100%"), las=TRUE,

cex.axis=0.9*axis.size)

axis(2, at=seq(0,1, by=0.2), lab=c("0%", "20%", "40%", "60%", "80%", "100%"), las=TRUE,

cex.axis=0.9*axis.size)

points(x=c(0.02, 0.68, 0.99, 0.99), y=c(0.02, 0.38, 0.55, 0.75), pch=19, cex=1 )

text(x=0.09, y=0.03, labels="control", cex=1)

text(x=0.52, y=0.38, labels="0.001% PPF nets", cex=1)

text(x=0.85, y=0.55, labels="0.01% PPF nets", cex=1)

text(x=0.85, y=0.75, labels="0.1% PPF nets", cex=1)

arrows(x0=0.6, y0=0.02, x1=0.99, y1=0.02, length=0.1, lwd=2)

arrows(x1=0.6, y1=0.02, x0=0.99, y0=0.02, length=0.1, lwd=2)

text(x=0.8, y=0.06, labels="PPF surfaces", cex=1.25)

text(x=0.05, y=0.95, labels="D", cex=1.5)

#############

## ##

## LEGEND ##

## ##

#############

par(mar=c(4,10,0,10))

par(mgp=c(2.25,0.75,0))

plot( x=seq(from=eggs_min, to=eggs_max, length=length(colours)), y=rep(0.5, length(colours)),

col=colours, pch=15, cex=10,

xaxt="n", yaxt="n", xaxs="i", yaxs="i", bty="n",

xlab = expression(paste( "E"^"SS", "/E"^"RR", " : ratio of susceptible to resistant eggs")), ylab="",

ylim=c(0,1), xlim=c(eggs_min, eggs_max),

cex.lab=lab.size )

axis(1, at=c(0,0.5,1,1.0,1.5,2), lab=c(0,0.5,1,1.0,1.5,2), las=TRUE,

cex.axis=0.9*axis.size)

dev.off()

##########################################################

##########################################################

## ##

## ##### #### #### ## ## ##### ##### ### #### ##

## ## ## ## ## ## ## ## ## ## ## ##

## #### ## ## ### ## ## ##### #### ### ### ##

## ## ## ## ## ## ## ## ## ## ## ## ##

## ## #### #### #### ## ## ##### ### #### ##

## ##

##########################################################

##########################################################

step_size <- 0.001

#################

## ##

## SIMULATIONS ##

## PANEL A ##

## ##

#################

fecund_seq <- seq(from=0, to=1-step_size, by=step_size) ## sequence for redcution in fecundity

life_seq <- seq(from=0, to=1-step_size, by=step_size) ## sequence for redcution in life expectancy

###################################################################

## Test parameter spce to see whether SS or RR mosquitoes oviposit more eggs

eggs_mat_30 <- matrix(NA, nrow=length(fecund_seq), ncol=length(life_seq))

for(i in 1:length(fecund_seq)){

for(j in 1:length(life_seq)){

eggs_mat_30[i,j] <- eggs_ratio( 1-fecund_seq[i], 1-life_seq[j], 0.3 )

}

}

SR_divide_30 <- rep(NA, length(fecund_seq))

for(i in 1:length(SR_divide_30)){

SR_divide_30[i] <- life_seq[which.min((eggs_mat_30[i,]-1)^2)]

}

#################

## ##

## SIMULATIONS ##

## PANEL B ##

## ##

#################

fecund_seq <- seq(from=0, to=1-step_size, by=step_size) ## sequence for redcution in fecundity

life_seq <- seq(from=0, to=1-step_size, by=step_size) ## sequence for redcution in life expectancy

###################################################################

## Test parameter spce to see whether SS or RR mosquitoes oviposit more eggs

eggs_mat_80 <- matrix(NA, nrow=length(fecund_seq), ncol=length(life_seq))

for(i in 1:length(fecund_seq)){

for(j in 1:length(life_seq)){

eggs_mat_80[i,j] <- eggs_ratio( 1-fecund_seq[i], 1-life_seq[j], 0.8 )

}

}

SR_divide_80 <- rep(NA, length(fecund_seq))

for(i in 1:length(SR_divide_80)){

SR_divide_80[i] <- life_seq[which.min((eggs_mat_80[i,]-1)^2)]

}

################################################

## Create colour gradient

Ncol = 1000

eggs_max <- 2 # max( eggs_mat1, eggs_mat2)

eggs_min <- 0 # min( eggs_mat1, eggs_mat2)

N_red <- round( Ncol*( 1-eggs_min )/( eggs_max - eggs_min ) )

N_green <- round( Ncol*( eggs_max-1 )/( eggs_max - eggs_min ) )

col_red_yellow <- rainbow(N_red, start=0, end=1/6)

col_yellow_green <- rainbow(N_green, start=1/6, end=2/6)

colours <- c(col_red_yellow, col_yellow_green)

tiff(file="Figure_S3.tif", width=20, height=12, units="cm", res=500)

lay.mat <- rbind( c(1,2), c(3,3) )

layout(lay.mat, heights=c(1,0.2))

layout.show(3)

par(mar=c(3,3,3,1.0))

par(mgp=c(2,0.75,0))

main.size = 1.1

axis.size = 1

lab.size = 1.25

line.size = 2

#############

## ##

## PANEL A ##

## ##

##################################################################

## Comparison of egg reduction vs. itn coverage parameter space ##

## for regions of suppression of resistance ##

##################################################################

par(mar=c(4,4,3,1))

par(mgp=c(2.25,0.75,0))

##########################

## Plot output

image( eggs_mat_30,

xlab="Reduction in fecundity", ylab="Reduction in life expectancy",

main=expression(paste( "E"^"SS", "/E"^"RR", " : lethality vs fecundity (30% ITN coverage)")),

col=colours, zlim=c(eggs_min, eggs_max),

xaxt="n", yaxt="n", xaxs="i", yaxs="i",

cex.main=main.size, cex.lab=lab.size, cex.axis=axis.size )

points(x=fecund_seq, y=SR_divide_30, type='l', lty="dashed", col="grey", lwd=2)

axis(1, at=seq(0,1, by=0.2), lab=c("0%", "20%", "40%", "60%", "80%", "100%"), las=TRUE,

cex.axis=0.9*axis.size)

axis(2, at=seq(0,1, by=0.2), lab=c("0%", "20%", "40%", "60%", "80%", "100%"), las=TRUE,

cex.axis=0.9*axis.size)

points(x=c(0.02, 0.68, 0.99, 0.99), y=c(0.02, 0.38, 0.55, 0.75), pch=19, cex=1 )

text(x=0.09, y=0.03, labels="control", cex=0.8)

text(x=0.52, y=0.38, labels="0.001% PPF nets", cex=0.8)

text(x=0.85, y=0.55, labels="0.01% PPF nets", cex=0.8)

text(x=0.85, y=0.75, labels="0.1% PPF nets", cex=0.8)

arrows(x0=0.6, y0=0.02, x1=0.99, y1=0.02, length=0.1, lwd=2)

arrows(x1=0.6, y1=0.02, x0=0.99, y0=0.02, length=0.1, lwd=2)

text(x=0.8, y=0.06, labels="PPF surfaces", cex=1.0)

text(x=0.05, y=0.95, labels="A", cex=1.5)

#############

## ##

## PANEL B ##

## ##

####################################################################

## Comparison of egg reduction vs. life reduction parameter space ##

## for regions of suppression of resistance ##

####################################################################

par(mar=c(4,4,3,1))

par(mgp=c(2.25,0.75,0))

#########################

## Plot output

image( eggs_mat_80,

xlab="Reduction in fecundity", ylab="Reduction in life expectancy",

main=expression(paste( "E"^"SS", "/E"^"RR", " : lethality vs fecundity (80% ITN coverage)")),

col=colours, zlim=c(eggs_min, eggs_max),

xaxt="n", yaxt="n", xaxs="i", yaxs="i",

cex.main=main.size, cex.lab=lab.size, cex.axis=axis.size )

points(x=fecund_seq, y=SR_divide_80, type='l', lty="dashed", col="grey", lwd=2)

axis(1, at=seq(0,1, by=0.2), lab=c("0%", "20%", "40%", "60%", "80%", "100%"), las=TRUE,

cex.axis=0.9*axis.size)

axis(2, at=seq(0,1, by=0.2), lab=c("0%", "20%", "40%", "60%", "80%", "100%"), las=TRUE,

cex.axis=0.9*axis.size)

points(x=c(0.02, 0.68, 0.99, 0.99), y=c(0.02, 0.38, 0.55, 0.75), pch=19, cex=1 )

text(x=0.09, y=0.03, labels="control", cex=0.8)

text(x=0.52, y=0.38, labels="0.001% PPF nets", cex=0.8)

text(x=0.85, y=0.55, labels="0.01% PPF nets", cex=0.8)

text(x=0.85, y=0.75, labels="0.1% PPF nets", cex=0.8)

arrows(x0=0.6, y0=0.02, x1=0.99, y1=0.02, length=0.1, lwd=2)

arrows(x1=0.6, y1=0.02, x0=0.99, y0=0.02, length=0.1, lwd=2)

text(x=0.8, y=0.06, labels="PPF surfaces", cex=1.0)

text(x=0.05, y=0.95, labels="B", cex=1.5)

#############

## ##

## LEGEND ##

## ##

#############

par(mar=c(4,10,0,10))

par(mgp=c(2.0,0.5,0))

plot( x=seq(from=eggs_min, to=eggs_max, length=length(colours)), y=rep(0.5, length(colours)),

col=colours, pch=15, cex=10,

xaxt="n", yaxt="n", xaxs="i", yaxs="i", bty="n",

xlab = expression(paste( "E"^"SS", "/E"^"RR", " : ratio of susceptible to resistant eggs")), ylab="",

ylim=c(0,1), xlim=c(eggs_min, eggs_max),

cex.lab=lab.size )

axis(1, at=c(0,0.5,1,1.0,1.5,2), lab=c(0,0.5,1,1.0,1.5,2), las=TRUE,

cex.axis=0.9*axis.size)

dev.off()
