# Supplementary material for: Negative Cross Resistance Mediated by Co-Treated Bed Nets: A Potential Means of Restoring Pyrethroid-Susceptibility to Malaria Vectors
Source: PLoS One. 2014 May 1;9(5):e95640. doi: 10.1371/journal.pone.0095640 (PMC4006834; doi:10.1371/journal.pone.0095640)
Supplement: R Code S2 — Code is for Figure 2. (DOCX) [file pone.0095640.s008.docx]

########################################################################################################

########################################################################################################

## Supporting File: R code for Figure 2 (main text) ##

## ##

## ##

## Please feel free to use and modify with proper citation. ##

## For any questions or comments, please contact ##

## ##

## ##

## Dr Michael White ##

## MRC Centre for Outbreak Analysis and Modelling ##

## Department of Infectious Disease Epidemiology ##

## Imperial College London ##

## m.white08@imperial.ac.uk ##

## ##

########################################################################################################

########################################################################################################

library(deSolve)

step_size <- 1

###############################################

## Larval model parameters from MCMC fitting ##

## process in White et al. ##

###############################################

d_E <- 6.64 ## duration of early instars

d_L <- 4.17 ## duration of late instars

d_P <- 0.64 ## duration of pupae

mu_E0 <- 0.034 ## death rate of early instars at low density

mu_L0 <- 0.035 ## death rate of early instars at low density

mu_P <- 0.25 ## death rate of pupae

mu_M0 <- 0.096 ## death rate of adult female mosquitoes - no nets

beta0 <- 21.19 ## daily oviposition rate per female mosquito

gamma <- 13.25 ## effect of density-dependence on early instars relative to late instars

delta <- 3 ## duration of gonotrophic cycle

eps0 <- beta0*(exp(delta*mu_M0)-1)/mu_M0 ## number of eggs per oviposition

mosq_gen <- d_E + d_L + d_P + 1/mu_M0 ## mosquito generation time

M_total_eq <- 1 ## equilibrium number of adult female mosquitoes

N_gono <- 10

M_gono_eq <- M_total_eq*exp( -(0:(N_gono-2))*mu_M0*delta )*( 1-exp(-mu_M0*delta) )

M_gono_eq <- c( M_gono_eq, M_total_eq-sum(M_gono_eq) )

##############################################

## Calculate the equilibrium carrying capacity

b_lambda <- gamma*mu_L0/mu_E0 - d_E/d_L + (gamma-1)*mu_L0*d_E

lambda <- -0.5*b_lambda + sqrt( 0.25*b_lambda^2 + gamma*beta0*mu_L0*d_E/(2*mu_E0*mu_M0*d_L*(1+d_P*mu_P)) )

KK <- M_total_eq*2*d_L*mu_M0*(1+d_P*mu_P)*gamma*(lambda+1)/( lambda/(mu_L0*d_E) - 1/(mu_L0*d_L) - 1 )

start_eq = c(2*lambda*mu_M0*d_L*(1+d_P*mu_P)*M_total_eq,

2*mu_M0*d_L*(1+d_P*mu_P)*M_total_eq,

2*d_P*mu_M0*M_total_eq,

M_gono_eq,

rep(0, N_gono) )

###########################################

## Genotype specific mosquito parameters

mu_M <- c(mu_M0, mu_M0, mu_M0)

beta <- c(beta0, beta0, beta0)

##############################################

## Other entomological parameters including ##

## those related to ITNs ##

##############################################

Q_0 <- 0.90 ## human blood index

phi <- 0.89 ## endophagy in bed

tau_1 <- 0.69 ## time spent searching for blood meal

tau_2 <- delta - tau_1 ## time spent resting and ovipositing

####################################################

## ITN resistance parameters taken from N'Guessan ##

## et al. ##

####################################################

h = 0.5 ## phenotypic heterozygosity: h=0 => SR=SS, h=1 => SR=RR

r_ss = 0.56

s_ss = 0.03

d_ss = 0.41

r_rr = 0.24

d_rr = 0.1

s_rr = 1 - r_rr - d_rr

r_sr = h*r_rr + (1-h)*r_ss

d_sr = h*d_rr + (1-h)*d_ss

s_sr = 1 - r_sr - d_sr

r_ITN <- c(r_ss, r_sr, r_rr)

d_ITN <- c(d_ss, d_sr, d_rr)

s_ITN <- c(s_ss, s_sr, s_rr)

PPF_PYR_sim <- function( itn_cov, P_eggs, P_life, ITN_on, PPF_on, sim_time ){

#########################################################

## ITN model - same as that described in Griffin et al ##

#########################################################

W <- 1 - Q_0*itn_cov*phi*(1 - s_ITN) ## probability of mosquito succeeding in feeding in a single attempt

Z <- Q_0*itn_cov*phi*r_ITN ## probability of mosquito repeating in a single attempt

f_ITN <- 1/( tau_1/(1-Z) + tau_2 ) ## rate at which mosquito feeds

p_10 <- exp(-mu_M*tau_1) ## probability of mosquito dying during feeding at zero ITN coverage

p_2 <- exp(-mu_M*tau_2) ## probability of mosquito dying during resting and oviposition

p_1 <- p_10*W/(1-Z*p_10) ## probability of mosquito dying during feeding

mu_M_ITN <- - f_ITN*log(p_1*p_2) ## daily mosquito mortality

#########################################

## Calculate the reduced oviposition rate

beta_ITN <- eps0*mu_M_ITN/( exp(mu_M_ITN/f_ITN) - 1 )

#########################################

## Calculate PPF model parameters

P_ppf = Q_0*itn_cov*phi*(r_ITN + s_ITN)

P_ppf[1] = 0

P_ppf_t = rep(0, 3)

mu_M_ppf <- mu_M/P_life

p_10_ppf <- exp(-mu_M_ppf*tau_1) ## probability of mosquito dying during feeding at zero ITN coverage

p_2_ppf <- exp(-mu_M_ppf*tau_2) ## probability of mosquito dying during resting and oviposition

p_1_ppf <- p_10*W/(1-Z*p_10_ppf) ## probability of mosquito dying during feeding

mu_M_ppf_ITN <- - f_ITN*log(p_1_ppf*p_2_ppf)

##mu_M_ppf_ITN <- mu_M_ITN/P_life

beta_ppf <- P_eggs*beta

beta_ppf_ITN <- P_eggs*eps0*mu_M_ppf_ITN/( exp(mu_M_ppf_ITN/f_ITN) - 1 )

############################################

## ODE larval model based on White et al

vector.par <- c()

times <- seq(from=0, to=sim_time, by=step_size)

################################################

## Set up initial conditions for differential equations

init_freq_sr <- 1e-5

init_freq <- c(1-2*init_freq_sr, init_freq_sr, init_freq_sr) ## initial frequency of resistance alleles

start = rep(0, 9+6*N_gono)

for(i in 1:3){

start[1+i-1] = start_eq[1]*init_freq[i]

start[4+i-1] = start_eq[2]*init_freq[i]

start[7+i-1] = start_eq[3]*init_freq[i]

}

start[10:(9+N_gono)] <- start_eq[4:(3+N_gono)]*init_freq[1] ## M1

start[(10+N_gono):(9+2*N_gono)] <- start_eq[4:(3+N_gono)]*init_freq[2] ## M2

start[(10+2*N_gono):(9+3*N_gono)] <- start_eq[4:(3+N_gono)]*init_freq[3] ## M3

start[(10+3*N_gono):(9+4*N_gono)] <- 0 ## M1_ppf

start[(10+4*N_gono):(9+5*N_gono)] <- 0 ## M2_ppf

start[(10+5*N_gono):(9+6*N_gono)] <- 0 ## M3_ppf

larval.model = function(t, x, vector.par){

with(as.list(vector.par),{

dE = rep(0,3)

dL = rep(0,3)

dP = rep(0,3)

dM1 = rep(0,N_gono)

dM2 = rep(0,N_gono)

dM3 = rep(0,N_gono)

dM1_ppf = rep(0,N_gono)

dM2_ppf = rep(0,N_gono)

dM3_ppf = rep(0,N_gono)

E = x[1:3]

L = x[4:6]

P = x[7:9]

M1 = x[10:(9+N_gono)]

M2 = x[(10+N_gono):(9+2*N_gono)]

M3 = x[(10+2*N_gono):(9+3*N_gono)]

M1_ppf = x[(10+3*N_gono):(9+4*N_gono)]

M2_ppf = x[(10+4*N_gono):(9+5*N_gono)]

M3_ppf = x[(10+5*N_gono):(9+6*N_gono)]

######################################

## PPF contact, birth and death rates

beta_t <- beta

mu_M_t <- mu_M

beta_ppf_t <- beta

mu_M_ppf_t <- mu_M

if( t > ITN_on ){

beta_t <- beta_ITN

mu_M_t <- mu_M_ITN

beta_ppf_t <- beta_ITN

mu_M_ppf_t <- mu_M_ITN

}

if( t > PPF_on ){

beta_ppf_t <- beta_ppf_ITN

mu_M_ppf_t <- mu_M_ppf_ITN

P_ppf_t = P_ppf

}

######################################

## genetic mixing

mm = c( sum(M1), sum(M2), sum(M3) )/sum(M1+M2+M3)

omega <- rep(NA, 3)

omega_ppf <- rep(NA, 3)

omega[1] <- sum(M1[-1])*beta_t[1]*(mm[1]+0.5*mm[2]) + sum(M2[-1])*beta_t[2]*(0.5*mm[1]+0.25*mm[2])

omega[2] <- sum(M1[-1])*beta_t[1]*(0.5*mm[2]+mm[3]) + sum(M2[-1])*beta_t[2]*(0.5*mm[1]+0.5*mm[2]+0.5*mm[3]) + sum(M3[-1])*beta_t[3]*(0.5*mm[2]+mm[3])

omega[3] <- sum(M2[-1])*beta_t[2]*(0.25*mm[2]+0.5*mm[3]) + sum(M3[-1])*beta_t[3]*(0.5*mm[2]+mm[3])

omega_ppf[1] <- sum(M1_ppf[-1])*beta_ppf_t[1]*(mm[1]+0.5*mm[2]) + sum(M2_ppf[-1])*beta_ppf_t[2]*(0.5*mm[1]+0.25*mm[2])

omega_ppf[2] <- sum(M1_ppf[-1])*beta_ppf_t[1]*(0.5*mm[2]+mm[3]) + sum(M2_ppf[-1])*beta_ppf_t[2]*(0.5*mm[1]+0.5*mm[2]+0.5*mm[3]) + sum(M3_ppf[-1])*beta_ppf_t[3]*(0.5*mm[2]+mm[3])

omega_ppf[3] <- sum(M2_ppf[-1])*beta_ppf_t[2]*(0.25*mm[2]+0.5*mm[3]) + sum(M3_ppf[-1])*beta_ppf_t[3]*(0.5*mm[2]+mm[3])

######################################

## Differential equation model

for(i in 1:3){

dE[i] = omega[i] + omega_ppf[i] - mu_E0*( 1 + sum(E+L)/KK )*E[i] - E[i]/d_E

dL[i] = E[i]/d_E - mu_L0*( 1+gamma*sum(E+L)/KK )*L[i] - L[i]/d_L

dP[i] = L[i]/d_L - mu_P*P[i] - P[i]/d_P

}

dM1[1] = 0.5*P[1]/d_P - mu_M_t[1]*M1[1] - M1[1]*f_ITN[1]

dM1[2:(N_gono-1)] = (1-P_ppf_t[1])*M1[1:(N_gono-2)]*f_ITN[1] - mu_M_t[1]*M1[2:(N_gono-1)] - M1[2:(N_gono-1)]*f_ITN[1]

dM1[N_gono] = (1-P_ppf_t[1])*M1[N_gono-1]*f_ITN[1] - mu_M_t[1]*M1[N_gono]

dM2[1] = 0.5*P[2]/d_P - mu_M_t[2]*M2[1] - M2[1]*f_ITN[2]

dM2[2:(N_gono-1)] = (1-P_ppf_t[2])*M2[1:(N_gono-2)]*f_ITN[2] - mu_M_t[2]*M2[2:(N_gono-1)] - M2[2:(N_gono-1)]*f_ITN[2]

dM2[N_gono] = (1-P_ppf_t[2])*M2[N_gono-1]*f_ITN[2] - mu_M_t[2]*M2[N_gono]

dM3[1] = 0.5*P[3]/d_P - mu_M_t[3]*M3[1] - M3[1]*f_ITN[3]

dM3[2:(N_gono-1)] = (1-P_ppf_t[3])*M3[1:(N_gono-2)]*f_ITN[3] - mu_M_t[3]*M3[2:(N_gono-1)] - M3[2:(N_gono-1)]*f_ITN[3]

dM3[N_gono] = (1-P_ppf_t[3])*M3[N_gono-1]*f_ITN[3] - mu_M_t[3]*M3[N_gono]

dM1_ppf[1] = 0

dM1_ppf[2:(N_gono-1)] = P_ppf_t[1]*M1[1:(N_gono-2)]*f_ITN[1] + M1_ppf[1:(N_gono-2)]*f_ITN[1] - mu_M_ppf_t[1]*M1_ppf[2:(N_gono-1)] - M1_ppf[2:(N_gono-1)]*f_ITN[1]

dM1_ppf[N_gono] = P_ppf_t[1]*M1[N_gono-1]*f_ITN[1] + M1_ppf[N_gono-1]*f_ITN[1] - mu_M_ppf_t[1]*M1_ppf[N_gono]

dM2_ppf[1] = 0

dM2_ppf[2:(N_gono-1)] = P_ppf_t[2]*M2[1:(N_gono-2)]*f_ITN[2] + M2_ppf[1:(N_gono-2)]*f_ITN[2] - mu_M_ppf_t[2]*M2_ppf[2:(N_gono-1)] - M2_ppf[2:(N_gono-1)]*f_ITN[2]

dM2_ppf[N_gono] = P_ppf_t[2]*M2[N_gono-1]*f_ITN[2] + M2_ppf[N_gono-1]*f_ITN[2] - mu_M_ppf_t[2]*M2_ppf[N_gono]

dM3_ppf[1] = 0

dM3_ppf[2:(N_gono-1)] = P_ppf_t[3]*M3[1:(N_gono-2)]*f_ITN[3] + M3_ppf[1:(N_gono-2)]*f_ITN[3] - mu_M_ppf_t[3]*M3_ppf[2:(N_gono-1)] - M3_ppf[2:(N_gono-1)]*f_ITN[3]

dM3_ppf[N_gono] = P_ppf_t[3]*M3[N_gono-1]*f_ITN[3] + M3_ppf[N_gono-1]*f_ITN[3] - mu_M_ppf_t[3]*M3_ppf[N_gono]

list(c(dE, dL, dP, dM1, dM2, dM3, dM1_ppf, dM2_ppf, dM3_ppf))

})

}

larval.sim = as.data.frame(lsoda(y=start, times=times, func=larval.model, parm=vector.par,

atol=1e-8, rtol=1e-8))

mosq <- cbind( larval.sim[,1],

rowSums( larval.sim[11:(10+N_gono)]),

rowSums( larval.sim[(11+N_gono):(10+2*N_gono)]),

rowSums( larval.sim[(11+2*N_gono):(10+3*N_gono)]),

rowSums( larval.sim[(11+3*N_gono):(10+4*N_gono)]),

rowSums( larval.sim[(11+4*N_gono):(10+5*N_gono)]),

rowSums( larval.sim[(11+5*N_gono):(10+6*N_gono)]) )

colnames(mosq) <- c("time", "SS", "SR", "RR", "SS_ppf", "SR_ppf", "RR_ppf")

prev <- mosq[,2:7]/rowSums(mosq[,2:7])

prev <- cbind( rowSums(prev[,c(1,4)]), rowSums(prev[,c(2,3,5,6)]) )

prev <- cbind( times, prev )

colnames(prev) <- c("time", "S", "R")

XX <<- larval.sim

list( mosq, prev )

}

############################################

## Plot output

base_ITN <- PPF_PYR_sim( itn_cov=0.5, P_eggs=1, P_life=1,

ITN_on=200, PPF_on=10000, sim_time=3000 )

base_ITN_0.001 <- PPF_PYR_sim( itn_cov=0.5, P_eggs=0.32, P_life=0.62,

ITN_on=200, PPF_on=1500, sim_time=3000 )

#base_IRS <- PPF_PYR_sim( itn_cov=0.5, P_eggs=0.40, P_life=1,

# ITN_on=200, PPF_on=1500, sim_time=3000 )

#base <- PPF_PYR_sim( itn_cov=0.0, P_eggs=1, P_life=1,

# ITN_on=10000, PPF_on=10000, sim_time=3000 )

#base_ITN_0.01 <- PPF_PYR_sim( itn_cov=0.5, P_eggs=0.40, P_life=1,

# ITN_on=200, PPF_on=1500, sim_time=3000 )

#base_ITN_0.1 <- PPF_PYR_sim( itn_cov=0.5, P_eggs=0, P_life=0.25,

# ITN_on=200, PPF_on=1500, sim_time=3000 )

############################################

############################################

## ##

## ##### #### #### ## ## ##### ##### ##

## ## ## ## ## ## ## ## ## ##

## #### ## ## ### ## ## ##### #### ##

## ## ## ## ## ## ## ## ## ## ##

## ## #### #### #### ## ## ##### ##

## ##

############################################

############################################

tiff(file="Figure2.tif", width=20, height=12, units="cm", res=500)

lay.mat <- rbind( c(1, 2), c(3, 3) )

layout(lay.mat, heights=c(12,1))

layout.show(3)

main.size = 1.5

axis.size = 0.8

lab.size = 1

line.size = 2

par(mar=c(3,3,2,2))

par(mgp=c(1.6,0.8,0))

############

## PANEL 1

plot(x=base_ITN[[2]][,1], y=base_ITN[[2]][,2],

type='l', lwd=line.size, col="green",

ylim=c(0,1.1), xlim=c(0,120*mosq_gen),

xlab="", ylab="prevalence of resistance", main="Prevalence of resistance",

xaxt="n", xaxs="i",

cex.main=main.size, cex.lab=lab.size, cex.axis=axis.size )

axis(1, at=seq(from=0, to=120*mosq_gen, by=20*mosq_gen), lab=seq(from=0, to=120, by=20) )

title(xlab="mosquito generation")

points(x=base_ITN[[2]][,1], y=base_ITN[[2]][,3],

type='l', lwd=line.size, col="red" )

points(x=base_ITN_0.001[[2]][,1], y=base_ITN_0.001[[2]][,2],

lty="longdash", type='l', lwd=line.size, col="green" )

points(x=base_ITN_0.001[[2]][,1], y=base_ITN_0.001[[2]][,3],

lty="longdash", type='l', lwd=line.size, col="red" )

arrows(x0=200, y0=1.1, x1=200, y1=1.01, lwd=1, length=0.1)

text(x=450, y=1.08, cex=0.9, label="PYR nets")

arrows(x0=1500, y0=1.1, x1=1500, y1=1.01, lwd=1, length=0.1)

text(x=1870, y=1.08, cex=0.9, label="PYR/PPF nets")

text(x=75, y=1.11, cex=1.2, label="A")

############

## PANEL 2

par(mgp=c(1.6,0.8,0))

plot(x=base_ITN[[1]][,1], y=rowSums(base_ITN[[1]][,c(2,5)]),

type='l', lwd=line.size, col="green",

ylim=c(0,1.1), xlim=c(0,120*mosq_gen),

xlab="", ylab="mosquito numbers", main="Mosquito numbers",

xaxt="n", xaxs="i", yaxt="n",

cex.main=main.size, cex.lab=lab.size, cex.axis=axis.size )

axis(1, at=seq(from=0, to=120*mosq_gen, by=20*mosq_gen), lab=seq(from=0, to=120, by=20))

title(xlab="mosquito generation")

axis(2, at=seq(from=0, to=1, by=0.25), lab=seq(from=0, to=1000, by=250) )

points(x=base_ITN[[1]][,1], y=rowSums(base_ITN[[1]][,c(3,4,6,7)]),

type='l', lwd=line.size, col="red" )

points(x=base_ITN_0.001[[1]][,1], y=rowSums(base_ITN_0.001[[1]][,c(2,5)]),

lty="longdash", type='l', lwd=line.size, col="green" )

points(x=base_ITN_0.001[[1]][,1], y=rowSums(base_ITN_0.001[[1]][,c(3,4,6,7)]),

lty="longdash", type='l', lwd=line.size, col="red" )

arrows(x0=200, y0=1.1, x1=200, y1=1.01, lwd=1, length=0.1)

text(x=450, y=1.08, cex=0.9, label="PYR nets")

arrows(x0=1500, y0=1.1, x1=1500, y1=1.01, lwd=1, length=0.1)

text(x=1870, y=1.08, cex=0.9, label="PYR/PPF nets")

text(x=75, y=1.11, cex=1.2, label="B")

par(mgp=c(0.75,1,0))

#################

## Legend

oldMar <- par(mar = c(0,0,0,0))

plot.new()

legend(x='center', legend = c("PYR treated nets (SS)", "PYR treated nets (SR + RR)",

"PYR/PPF co-treated nets (SS)", "PYR/PPF co-treated nets (SR + RR)" ),

col = c("green", "red", "green", "red"),

ncol=2,

pch=19, lwd = 2, pt.cex=0, cex=1.1, bty="n", lty=c(1,1,3,3) )

par(oldMar)

dev.off()
