# Supplementary material for: Negative Cross Resistance Mediated by Co-Treated Bed Nets: A Potential Means of Restoring Pyrethroid-Susceptibility to Malaria Vectors
Source: PLoS One. 2014 May 1;9(5):e95640. doi: 10.1371/journal.pone.0095640 (PMC4006834; doi:10.1371/journal.pone.0095640)
Supplement: Text S1 — Contains Table S1, parameters describing the behaviour and life history of An. gambiae s. s. mosquitoes. Table S2, parameters describing interactions between a mosquito and an insecticide-treated net. Table S3, notation, definition and values of the variables and parameters for the model of A. gambiae population dynamics. All parameter values are taken from White et al [2]. (DOCX) [file pone.0095640.s009.docx]

**Supporting Information**

Negative cross resistance mediated by co-treated bed nets: a potential means of restoring pyrethroid-susceptibility to malaria vectors.

Michael T. White^1^, Dickson Lwetoijera^2^, John Marshall^1^, Geoffrey Caron-Lormier^3^, David A. Bohan^4^, Ian Denholm^5^, Gregor J Devine^6^*

^1^ MRC Centre for Outbreak Analysis and Modelling, Imperial College, London W21PG, UK.

^2^ Ifakara Health Institute, PO Box 53, Ifakara, Tanzania.

^3^ University of Nottingham, Sutton Bonington, Leicestershire LE125RD, UK

^4^ INRA, UMR 1347 Agroécologie, Pôle ECOLDUR, 17 rue Sully, 21065 Dijon, France.

^5^ University of Hertfordshire, Hatfield, Hertfordshire, AL109AB, UK

^6^ QIMR Berghofer Medical Research Institute, Brisbane 4006, Australia.

# Characteristics of the vector population

We model a population of *Anopheles* *gambiae* s. s. mosquitoes whose behaviour and life history are described by the parameters outlined in Table S1.

# Effect of vector control on mosquito populations

We describe models for the effects of conventional pyrethroid-treated nets and co-treated pyrethroid/pyriproxyfen (PPF) nets on *An. gambiae* s. s. mosquitoes. Although we focus on a strategy where PPF is co-applied to pyrethroid-treated nets, the model is also applicable to scenarios where the same chemical combination is applied to walls as an indoor residual spray.

## *2.1 Pyrethroid treated nets*

We adapt an existing model of the effects of insecticide treated bed nets (ITNs) on *Anopheles* mosquitoes [6,7]. Parameters describing the effect of ITNs on the mosquito’s lifecycle are detailed in Table S2. Figure S1 depicts a flow chart of the interaction between a mosquito and an ITN.

The rate at which mosquitoes feed on humans *a*, can be calculated in terms of the rate at which a female mosquito completes a feeding cycle *f* = 1/δ, and the proportion of blood meals taken on humans *Q*_0_:

$$a=fQ_{0}$$

From the flow chart in Figure S1, with ITN coverage *C*, the probability that a surviving mosquito succeeds in feeding during a single attempt is

where ϕ, and *s* are as defined in Tables S1 and S2. The probability of a mosquito being forced to repeat its attempt to feed and begin a new search is

where *r* is the probability of a mosquito being repelled by a bed net. In the absence of ITNs the length of a feeding cycle is given by

$$\frac{1}{f}=\delta=\tau_{1}(0)+\tau_{2}$$

Increased ITN coverage will cause the mosquito to spend a longer time foraging for a blood meal. At ITN coverage *C*

$$\tau_{1}(C)=\tau_{1}(0)+z(C)\tau_{1}(C)=\frac{\tau_{1}(0)}{1-z(C)}$$

And therefore the length of a feeding cycle at coverage *C* is

$$\frac{1}{f(C)}=\delta(C)=\frac{\tau_{1}(0)}{1-z}+\tau_{2}$$

Increased ITN coverage will also reduce the probability of a mosquito surviving the foraging stage$p_{1}$.

$$p_{1}(C)=p_{1}(0)[w(C)+z(C)p_{1}(C)]=\frac{p_{1}(0)w(C)}{1-z(C)p_{1}(0)}$$

Thus the probability of a mosquito surviving one day is given by

$$p(C)=\left(p_{1}(C)p_{2}\right)^{f(C)}=\left(\frac{p_{1}(0)w(C)p_{2}}{1-z(C)p_{1}(0)}\right)^{f(C)}$$

where$p_{2}$is the probability of surviving resting. The mosquito mortality can then be calculated as

$$\mu_{M,ITN}(C)=-\log p(C)$$

## *2.2. Exposure to pyriproxyfen at treated surfaces*

We assume that the transfer of PPF from a treated surface to a female mosquito increases her mortality to *µ_M_*_,PPF_ ≥ *µ_M_* and decreases the number of eggs per oviposition cycle to ε_PPF_ ≤ ε. We assume that pyrethroid-susceptible mosquitoes are never subjected to the effects of PPF as they are killed, irritated or repelled upon exposure to pyrethroid. We assume that pyrethroid-resistant mosquitoes are not repelled by pyrethroid on the net. Instead they will either land on the net and be diverted by the physical barrier of the net, or land on the net and successfully feed through a hole. In both cases, they will be exposed to PPF. During each feeding attempt, the probability that a resistant mosquito is exposed to PPF is

1. **Reproductive fitness**

We denote *E*^SS^ to be the expected number of eggs that a pyrethroid-susceptible female mosquito will oviposit over her lifetime. A female mosquito will survive the first gonotrophic cycle of length δ days with probability $e^{-\mu_{M,\text{ITN}}\delta}$ and oviposit ε eggs. The mosquito will survive each subsequent gonotrophic cycle with probability$e^{-\mu_{M,\text{ITN}}\delta}$. The expected number of eggs oviposited over her lifetime will then be

$$E^{SS}=\left(\varepsilon e^{-\mu_{M,\mathrm{ITN}} \text{ } \delta}+\varepsilon e^{-2\mu_{\text{M,ITN}} \text{ } \delta}+...\right)=\frac{\varepsilon}{e^{\mu_{\text{M,ITN}} \text{ } \delta}-1}$$

Pyrethroid resistant (RR) mosquitoes will contact PPF on co-treated surfaces with probability *p*_PPF_ every time they take a blood meal. We denote *M_n_* to be the number of mosquitoes having completed *n* gonotrophic cycles, and *M*_PPF_*_,n_* to be the number of mosquitoes having completed *n* cycles and been exposed to PPF. Figure S2 shows a flow chart from which the proportion of mosquitoes in each category can be calculated. Summing over all gonotrophic cycles gives the expected number of eggs oviposited by a pyrethroid resistant mosquito throughout her lifetime:

$$E^{RR}=\underbrace{\left(1-p_{\text{PPF}}\right)\frac{\varepsilon}{e^{\mu_{M,\text{ITN}}^{R}\delta^{R}}-1+p_{\text{PPF}}}}_{\text{eggs oviposited before PPF contact}}+\underbrace{p_{\text{PPF}}\frac{\varepsilon_{\text{PPF}}}{e^{\mu_{M,\text{ITN}}^{R}\delta^{R}}-1+p_{\text{PPF}}}\frac{1}{1-e^{-\mu_{M,\text{ITN-PPF}}^{R}\delta^{R}}}}_{\text{eggs oviposited after PPF contact}}$$

Figure S3 extends the results presented in Figure 1 of the main manuscript by comparing the reproductive fitness of pyrethroid susceptible and resistant mosquitoes when 30% and 80% ITN coverage is achieved. At 80% ITN coverage, treating nets with a 0.001% (w/v) solution of PPF is not enough to prevent the emergence of pyrethroid resistance.

# Mosquito population dynamics

The static model described in Section 3 (above) allows a comparison of reproductive fitness in terms of numbers of eggs oviposited by pyrethroid susceptible (SS) and resistant (RR) mosquitoes. Here we extend this model to incorporate mosquito population dynamics with variable proportions of SS, SR and RR mosquitoes. We adapt a previously published model [2] of the dynamics of the adult and juvenile stages of *Anopheles* mosquitoes to investigate the dynamics of mosquito populations in response to co-treated nets. The model is sequentially extended to incorporate: gonotrophic cycle tracking (Section 4.2 below), mixing of genotypes (Section 4.3 below) and exposure of adults to co-treated nets (Section 4.4 below).

The methods outlined below allow a comparison of the reproductive fitness of kdr homozygous susceptible (SS), heterozygous resistant (SR) and homozygous resistant (RR) mosquitoes in terms of the number of eggs oviposited by a dynamically mixing mosquito population. The model is continuous and deterministic and does not capture stochastic events. We do not attempt to model the stochastic emergence of *de novo* kdr resistance due to random mutations. Instead we begin with a small initial frequency of kdr resistant mosquitoes (1 RR individual per 100,000 mosquitoes). This challenge in modelling the stochastic initial emergence of resistance means that we cannot accurately predict time to emergence of resistance, even if the differences in reproductive fitness due to pyrethroid-resistance have been accurately characterized. Furthermore, we ignore the issue of immigration of resistant mosquitoes. Finally, we assume that heterozygous resistant mosquitoes have phenotypic properties intermediate between those of homozygous susceptible and homozygous resistant mosquitoes.

## *4.1. Modelled population dynamics*

We adapt a previously published model of the dynamics of the adult and juvenile stages of *Anopheles* mosquitoes [2]. Its parameters are described in Table S3. The aquatic part of the mosquito lifecycle consists of an egg stage, four larval instar stages and a pupal stage (the pre-imaginal stages). We group the eggs and the first two larval instars into the ‘early larval instar stage’ *E*. The third and fourth larval instars are grouped together as ‘late larval instars’ *L*. The pupal stage is denoted by *P* and the female adult mosquito stage by *M*. On average each female mosquito will lay β eggs per day, which will hatch into early instar larvae *E*. The number of eggs oviposited per day can be derived from the number of eggs per oviposition as follows

$$\beta=\varepsilon\frac{\mu_{M}}{e^{\delta\mu_{M}}-1}$$

Larvae will undergo density-dependent daily mortality at a rate which is assumed to depend on the density of both ‘early’ and ‘late’ instars. Larvae surviving the developmental period of *d_E_* days (where 1/*d_E_* is the rate of progression to the next stage) will develop into late instars *L*. These larvae will undergo density-dependent daily mortality at a rate $\mu_{L}^{0}\left(1+\gamma\frac{E+L}{K}\right)$during the *d_L_* days of development. K is the carrying capacity of the aquatic environment and determines the number of larvae and pupae, and hence the number of adult mosquitoes. Instars that survive development will become pupae at a rate 1/*d_L_* which are subjected to density independent mortality at a constant rate *µ_P_* throughout their *d_P_* days of development. It is assumed that half of all emerging adult mosquitoes are female. Male adult mosquitoes are ignored, only assuming there are enough males for successful mating with females. Emerging adult female mosquitoes will search for a blood meal and begin their gonotrophic cycle. Although adult mosquitoes senesce we make the simplifying assumption that they undergo constant daily mortality at rate *µ_M_*. The mosquito lifecycle can thus be described by the following set of continuous ordinary differential equations.

$$\begin{matrix} \frac{dE}{dt}=\beta M-\frac{E}{d_{E}}-\mu_{E,0}\left(1+\frac{E+L}{K}\right)E \\ \frac{dL}{dt}=\frac{E}{d_{E}}-\frac{L}{d_{L}}-\mu_{L,0}\left(1+\gamma\frac{E+L}{K}\right)L \\ \frac{dP}{dt}=\frac{L}{d_{L}}-\frac{P}{d_{P}}-\mu_{P}P \\ \frac{dM}{dt}=\frac{1}{2}\frac{P}{d_{P}}-\mu_{M}M \end{matrix}$$

We assume the mosquito generation time to be the expected lifespan of the aquatic and adult stages:

$$T_{M}=d_{E}+d_{L}+d_{P}+\frac{1}{\mu_{M}}$$

## *4.2. Mosquito population dynamics with tracking of gonotrophic cycle*

We next extend the model so that the number of completed gonotrophic cycles of adult female mosquitoes can be tracked. We denote *M_n_* to be the number of female mosquitoes that have completed *n* ≤ *N* gonotrophic cycles. We choose *N* = 10.

$$\begin{matrix} \frac{dE}{dt}=\beta\left(\sum_{n=1}^{N} M_{n}\right)-\frac{E}{d_{E}}-\mu_{E,0}\left(1+\frac{E+L}{K}\right)E \\ \frac{dL}{dt}=\frac{E}{d_{E}}-\frac{L}{d_{L}}-\mu_{L,0}\left(1+\gamma\frac{E+L}{K}\right)L \\ \frac{dP}{dt}=\frac{L}{d_{L}}-\frac{P}{d_{P}}-\mu_{P}P \\ \frac{dM_{0}}{dt}=\frac{1}{2}\frac{P}{d_{P}}-\mu_{M}M_{0}-\frac{M_{0}}{\delta} \\ \frac{dM_{n}}{dt}=\frac{M_{n-1}}{\delta}-\mu_{M}M_{n}-\frac{M_{n}}{\delta} \text{ }\left(1\leq n\leq N-1\right)\text{ } \\ \frac{dM_{N}}{dt}=\frac{M_{N-1}}{\delta}-\mu_{M}M_{N} \end{matrix}$$

## *Mosquito population dynamics with mixing of genotypes*

We next extend the model of mosquito population dynamics with tracking of gonotrophic cycles described in Section 4.2 (above) to include the genotype frequency of a single allele conferring *kdr* resistance. Let *E^k^*, *L^k^* , *P^k^* and *M^k^* denote the numbers of early instar larvae, late instar larvae, pupae and adult mosquitoes of genotype $k\in\{SS,SR,RR\}$. Let $m^{k}=\frac{M^{k}}{\sum_{j} M^{j}}$ denote the proportion of adult mosquitoes of genotype$k$. The mosquito population dynamics with mixing of genotypes is then described by:

## *Mosquito population dynamics in response to co-treated nets*

## We extend the model outlined in Section 4.3 (above) to incorporate the effects of pyrethroid/PPF co-treated nets. Figure S4 shows a compartmental representation of the life history of an *Anopheles* mosquito attempting to take blood meals from a human population protected by co-treated nets. The model is described by the large set of coupled ordinary differentials in equation 18. This model tracks 4 different life stages, *N* gonotrophic stage of adult mosquitoes, 3 kdr genotypes, and a class for PPF exposed adults. The R code for implementing this model and generating Figure 2 (main text) is provided in a supporting file (R code S2).

## (18)

## *The effect of heterozygous (SR) mosquitoes*

In Figure 2 of the main text it is assumed that heterozygous resistant mosquitoes (SR) have phenotypic behaviour intermediate between that of homozygous susceptible (SS) and homozygous resistant (RR) mosquitoes, i.e. the dominance coefficient *h* = 0.5. Here, we extend our analysis to consider the scenario where SR mosquitoes have the same phenotypic behaviour as RR mosquitoes (*h* = 1, Figure S5), and where SR mosquitoes have similar phenotypic behaviour to SS mosquitoes (*h* = 0.1, Figure S6). Under these conditions of bed net coverage and imposed fitness costs, pyrethroid resistance was not predicted to emerge over the timescale considered.

References

1. Molineaux L, Gramiccia G (1980) The Garki project: Research on the Epidemiology and Control of Malaria in the Sudan Savanna of West Africa. Geneva: WHO. 311 p.

2. White MT, Griffin JT, Churcher TS, Ferguson NM, Basáñez M, et al. (2011) Modelling the impact of vector control interventions on Anopheles gambiae population dynamics. Parasites and Vectors 4.

3. Killeen GF, McKenzie FE, Foy BD, Schieffelin C, Billingsley PF, et al. (2000) A simplified model for predicting malaria entomologic inoculation rates based on entomologic and parasitologic parameters relevant to control. American Journal Of Tropical Medicine And Hygiene 62: 535-544.

4. Dia I, Diop T, Rakotoarivony I, Kengne P, Fontenille D (2003) Bionomics of Anopheles gambiae Giles, An. arabiensis Patton, An. funestus Giles and An. nili (Theobald) (Diptera: Culicidae) and transmission of Plasmodium falciparum in a Sudano-Guinean zone (Ngari, Senegal). J Med Entomol 40: 279-283.

5. Githeko AK, Service MW, Mbogo CM, Atieli FK (1996) Resting behaviour, ecology and genetics of malaria vectors in large scale agricultural areas of Western Kenya. Parassitologia 38: 481-489.

6. Griffin JT, Hollingsworth TD, Okell LC, Churcher TS, White M, et al. (2010) Reducing Plasmodium falciparum Malaria Transmission in Africa: A Model-Based Evaluation of Intervention Strategies. PLoS Med 7.

7. Le Menach A, Takala S, McKenzie FE, Perisse A, Harris A, et al. (2007) An elaborated feeding cycle model for reductions in vectorial capacity of night-biting mosquitoes by insecticide-treated nets. Malaria Journal 6.

8. Curtis CF, Myamba J, Wilkes TJ (1996) Comparison of different insecticides and fabrics for anti-mosquito bed nets and curtains. Medical And Veterinary Entomology 10: 1-11.

9. Lines JD, Myamba J, Curtis CF (1987) Experimental Hut Trials Of Permethrin-Impregnated Mosquito Nets And Eave Curtains Against Malaria Vectors In Tanzania. Medical And Veterinary Entomology 1: 37-51.

10. Curtis CF, Miller JE, Hodjati MH, Kolaczinski JH, Kasumba I (1998) Can anything be done to maintain the effectiveness of pyrethroid-impregnated bed nets against malaria vectors? PhilTrans R Soc Lond B 353: 1769-1775.

11. Ohashi O, Nakada K, Ishiwatari T, Miyaguchi J, Shono Y, et al. (2012) Efficacy of pyriproxyfen treated nets in sterilizing and shortening the longevity of Anopheles gambaie (Diptera: Culicidae). Journal of Medical Entomology 49: 1052-1058.

12. Harris C, Lwetoijera DW, Dongus S, Matowo NS, Lorenz LM, et al. (2013) Sterilising effects of pyriproxyfen on Anopheles arabiensis and its potential use in malaria control. Parasites & Vectors 6.

Table S1

| **Parameter** | **Description** | **Value** | **Reference** |
| --- | --- | --- | --- |
| *µ_M_* | Baseline daily mosquito mortality | 0.096 day^-1^ | [1,2] |
| δ | Duration of gonotrophic cycle – increases with ITN coverage | 3 days | [3] |
| τ_1_ | Time spent seeking blood meal during gonotrophic cycle (a function that increases with ITN coverage) | 0.69 days | [3] |
| τ_2_ | Time spent resting during gonotrophic cycle (a constant) | 2.31 days | [3] |
| ε | Eggs per oviposition | 74 | [2] |
| *Q*_0_ | Human blood index – proportion of blood meals on humans | 0.92 | [4] |
| ϕ | Proportion of bites taken on humans when in bed indoors | 0.89 | [5] |

Table S2

| **Parameter** | **Description** | **Value** | | **Reference** |
| --- | --- | --- | --- | --- |
|  |  | pyrethroid resistance | | |
|  |  | susceptible | resistant |  |
| *C* | ITN coverage | – | – | – |
| *s* | Probability of successfully feeding with ITN | 0.03 | 0.66 | [8,9] |
| *r* | Probability of repeating cycle with ITN | 0.56 | 0.24 | [8,9] |
| *d* | Probability of dying with ITN | 0.41 | 0.10 | [9,10] |
| *w* | Probability of mosquito successfully surviving feeding attempt | eq (2) | | – |
| *z* | Probability of mosquito repeating | eq (3) | | – |
| *p_1_* | Probability of mosquito surviving foraging stage | $p_{1}=e^{-\mu_{M}\tau_{1}}$ | | – |
| *p_2_* | Probability of mosquito surviving resting stage | $p_{2}=e^{-\mu_{M}\tau_{2}}$ | | – |

Table S3

| **Parameter** | **Description** | **Unit** | **Value** |
| --- | --- | --- | --- |
| *d_E_* | Development time of early larval instars | days | 6.64 |
| *d_L_* | Development time of late larval instars | days | 3.72 |
| *d_P_* | Development time of pupae | days | 0.64 |
| *µ_E_*_,0_ | Per capita daily mortality rate of early instars (low density) | day^-1^ | 0.034 |
| *µ_L_*_,0_ | Per capita daily mortality rate of late instars (low density) | day^-1^ | 0.035 |
| *µ_P_* | Per capita daily mortality rate of pupae | day^-1^ | 0.25 |
| *µ_M_* | Per capita daily mortality rate of adult *An. gambiae* | day^-1^ | 0.096 |
| β | No. of eggs laid per day per mosquito | – | 21.19 |
| γ | Effect of density dependence on late instars relative to early instars | – | 13.25 |
| δ | Duration of gonotrophic cycle | days | 3 |
| ε | Number of eggs per oviposition per mosquito | – | 74 |
| *h* | Dominance coefficient – describes the phenotypic properties of heterozygous resistant mosquitoes |  | 0.5 |
| *T_M_* | Mosquito generation time | days | 21.5 |
